# Supplementary material for: A Circulating Signature of Tumour Hybrid Cells and Immune Checkpoints Predicts Metastatic Progression in Lung Cancer
Source: Int J Mol Sci. 2026 Feb 19;27(4):1994. doi: 10.3390/ijms27041994 (PMC12940895; doi:10.3390/ijms27041994)
Supplement: Supplementary file 1 [file ijms-4122726-supplementary.pdf › ijms-4122726-supplementary.pdf]

Manuscript: A Circulating Signature of Tumour Hybrid Cells and Immune Checkpoints Predicts Metastatic Progression in Lung Cancer

We would like to express our sincere gratitude to the reviewers and the editor for their thoughtful and constructive feedback, which has substantially enhanced the quality and clarity of our manuscript. In response, we have undertaken a comprehensive revision addressing all technical and conceptual concerns raised. For ease of reference, all modifications have been highlighted in the revised version using the Track Changes function.

#Section Managing Editor

According to our journal's rule, the correct order of sections is: 1. Introductions, 2. Results, 3. Discussion, 4. Materials and Methods, 5. Conclusions. Please adjust the section order accordingly, including reference, Table, and Figure numbers.

The editor's feedback was appreciated and consequently, the order of the sections was changed to comply with the journal's guidelines.

#Reviewer 1

Congratulations to the authors for the interesting predictive/prognostic model.

There are, however, some issues that should be reviewed:

• The Materials and Methods section does not specify the patient inclusion and exclusion criteria or the recruitment time frame.

We appreciate the reviewer's suggestion and fully agree with the comment. To address this point, the inclusion and exclusion criteria have been updated into the "Participant recruitment and eligibility criteria" section of **Material and Methods** (page 11).

Page 11: *"The inclusion criteria were based on a histopathological diagnosis of primary non-small cell lung cancer (specifically adenocarcinoma and squamous cell carcinoma) in patients requiring diagnostic or therapeutic surgical intervention by the Thoracic Surgery Department at University Hospital La Paz. Furthermore, eligible participants were required to have provided written informed consent for both biobank storage and the collection of peripheral blood samples during routine follow-up prior to surgery.*

Patients were excluded from the study if they presented with benign lesions, secondary lung metastases, or pulmonary neuroendocrine tumours. Furthermore, exclusion was applied to patients whose clinical status, such as those in palliative care or elderly patients with mobility issues, prevented sample collection without exceeding the standard of care. Finally, patients receiving postoperative follow-up at external healthcare facilities were excluded to prevent redundant clinical visits and unnecessary blood draws."

• The sample size is limited and heterogeneous, lacking details on important variables such as PD-L1 (tissue) expression, presence of target genetic alterations, TNM classification used, and systemic treatments (neoadjuvant, adjuvant, or perioperative) administered.

- We thank the reviewer for this thoughtful comment. Given the exploratory nature and limited size of the present cohort, our primary objective was to generate hypotheses and identify candidate immune populations associated with clinical outcomes, rather than to establish definitive predictive models. This limitation has now been explicitly addressed in the **Limitations of the study** section, where we also emphasise that future studies incorporating larger and independent cohorts will be essential of external validate these findings (page 20).

Page 21: "*It is important to note that the present study is subject to certain limitations, which must be considered when interpreting the results. The cohort size was modest, with 31 patients included, which limits statistical power and may increase the risk of overfitting in the predictive models, particularly in complex models such as those applied in this study.*"

Page 21: "*Consequently, further mechanistic work is required to elucidate the biological pathways connecting tumour–immune fusion events with systemic immune dysregulation.*"

- In relation to the proposed clinical and demographic variables of the patients in the study, we have extended **Table 1** (pages 5-7) to encompass all of them.

• Most patients are diagnosed with stage I disease (28 patients), while 6 present with stage III–IV. As this cohort largely reflects surgical cases, clarification is needed regarding the rationale for operating on a stage IV patient. Additionally, the follow-up period of 2.5 years may be insufficient for a population predominantly comprised of stage I disease.

We would like to express our gratitude to the reviewer for this valuable observation. The patient in stage IV underwent surgical intervention for diagnostic purposes, as less invasive procedures had failed to provide a definitive histopathological diagnosis. The procedure in question corresponded to a diagnostic pulmonary resection (atypical segmentectomy) performed to obtain adequate tissue for diagnosis. This clarification has been incorporated into the revised manuscript (**Results section**, Pages 4-5).

Pages 4-5: "Patients were categorised according to key clinical parameters, including tumour histology, anatomical site, TNM stage, relapse or progression status, metastatic involvement, prior cancer history, lymphovascular invasion, surgical approach, smoking status, and the presence of comorbid respiratory disease. Most patients were diagnosed at stage I disease, while a minority presented with stage III–IV. The single-stage IV patient underwent surgical intervention for diagnostic purposes, as prior less-invasive procedures had not yielded a definitive histopathological diagnosis."

On the other hand, we acknowledge the reviewer's concern regarding the follow-up duration. While the cohort was predominantly composed of stage I patients, all recorded deaths occurred within the first 2.5 years of follow-up. Therefore, survival analyses were conducted using a 2.5-year time follow-up, as extending the period would not modify the observed survival outcomes.

Page 7: "*Using the same patient clustering obtained in **Figure 1A**, metastatic status was overlaid to evaluate their distribution across clusters; however, this unsupervised framework did not discriminate metastatic from non-metastatic patients (**Figure 2A**).*

Survival analyses were restricted to a 2.5-year follow-up period, as all observed deaths occurred within this interval."

• The inclusion of two typical carcinoid cases is noted; since carcinoids are distinct entities and not classified as lung carcinomas, it is recommended that they be excluded from the analysis.

We would like to express our gratitude to the reviewer for highlighting this significant issue. We are in full agreement that typical carcinoid tumours represent a distinct pathological entity and are not classified as lung carcinomas. Consequently, the two patients with typical carcinoid tumours have been excluded from the study cohort. All statistical analyses have been repeated, and all figures and tables in the manuscript have been updated to reflect this change. The exclusion of these cases did not alter the overall conclusions of the study.

• Some designations such as "metastatic" require clarification, particularly whether this refers to relapse or to patients metastatic at diagnosis. Including a patient who was metastatic at baseline complicates interpretation, and exclusion of this case is advised. Please also define what is meant by "exitus"—it is unclear if all patients with progression have died.

We would like to express our gratitude to the reviewer for highlighting this significant issue. It has been agreed that the definition of clinical endpoints requires clarification. In our study, "metastatic" refers to the presence of distant metastasis (i.e. the dissemination of neoplastic cells to organs different from the primary tumour site) detected during follow-up. *Exitus* was defined as death from any cause during the follow-up period (up to 2.5 years); importantly, not all patients with relapse or disease progression deceased during follow-up.

We have now provided a comprehensive definition of these clinical considerations in the **Material and Methods** section, **'Clinical definitions'** (pages 12-13).

Pages 12-13: *Clinical definitions*

The following clinical definitions were applied to ensure consistent classification of disease status and outcomes throughout the study. Metastasis was defined as the dissemination of neoplastic cells to organs different from the primary tumour site, as confirmed by clinical and radiological assessment, either at the time of diagnosis or during follow-up (e.g., lymph nodes, bone, contralateral lung, or other distant organs). Relapse was defined as the reappearance of neoplastic disease after a documented period of clinical, radiological, and/or analytical remission following initial treatment (surgery, chemotherapy, radiotherapy and/or immunotherapy). The progression of the disease was defined as a deterioration in the patient's condition during the follow-up period, as evidenced by the enlargement of existing tumours and/or the emergence of new metastatic lesions, in the absence of a prior interval without disease. The term "exitus" was defined as death from any cause during the follow-up period.

Regarding patients presenting with distant metastasis at baseline, we have verified that such cases were excluded from analyses aimed at modelling incident metastatic progression, as they would not be suitable for predictive modelling of metastasis.

As explained in our next answer, it should be noted that this does not imply that all patients with progression died.

• For patients who experienced relapse or disease progression, please clarify which patients were. Specifically, indicate whether these cases were stage III patients, as such findings would align with the anticipated natural history of the disease.

We thank the reviewer for this relevant comment. Among the five patients who experienced relapse or progression, one was initially classified as stage I, one as stage II, two as stage III, and one as stage IV disease. Of these, two patients (one stage III and one stage IV) died during follow-up. These findings are consistent with the known biological heterogeneity of NSLC, where recurrence may occur across different stages, although the risk increases with more advanced disease. This information has been incorporated into the **Results** section of the revised manuscript to facilitate interpretation (Pages 4-5).

Pages 4-5: "*Patients were categorised according to key clinical parameters, including tumour histology, anatomical site, TNM stage, relapse or progression status, metastatic involvement, prior cancer history, lymphovascular invasion, surgical approach, smoking status, and the presence of comorbid respiratory disease. During the 2.5-year follow-up period, five patients experienced relapse or disease progression. Of these, one case had initially been classified as stage I, one as stage II, two as stage III, and one as stage IV disease. Two of these patients (one stage III and one stage IV) died during the follow-up period. Demographic and clinical characteristics of the cohort are summarised in Table 1.*"

We would like to once again express our sincere gratitude to the reviewer for their constructive feedback and valuable suggestions, which have helped us to improve the clarity and rigour of our manuscript. We hope that the revisions detailed above satisfactorily addressed the reviewer's concerns and that the revised version will now be deemed suitable for publication.

#Reviewer 2

The manuscript by Gonzalo Sáenz de Santa María-Diez and his co-authors describes a model for predicting lung cancer prognosis based on the presence of tumor hybrid cells and the levels of soluble immunoregulatory molecules in the blood. The paper is well-written and will be of interest to readers of the journal. Despite the small number of patients, the presented results could be an initial step and indication to continue the search for better, more significant results. Several parameters that are valuable for predicting metastatic dissemination and mortality could be incorporated into lab tests in the near future.

Comments:

• There is no description of tumor hybrid cell detection by spectral flow cytometry in the Materials and Methods section. Please add this chapter.

We thank the reviewer for this insightful observation and fully agree with the comment. This methodological apart has been incorporated into the main text, specifically in the **Materials and Methods** section (page 13). The staining panel antibody was described in the **Supplementary Table 1** and the gating strategy is shown in the **Supplementary Figure 3**.

Page 13: "*Sample processing and full-spectrum flow cytometry.*

Whole blood samples were collected in EDTA (ethylenediaminetetraacetic acid anticoagulated) tubes (Vacuette®, Greiner Bio One, Kremsmünster, Austria). Red blood cell lysis was performed using BD PharmLyse™ Lysing Buffer (BD Biosciences), yielding a leukocyte cell suspension, which was washed twice with phosphate-buffered saline (PBS). Cell viability was assessed using LIVE/DEAD™ Fixable Blue dye (Invitrogen). Subsequently, cells were incubated with an antibody panel detailed in Supplementary Table 1. The staining panel was designed to measure circulating tumour hybrid cells, gated as the CD14+ population expressing EpCAM+ and CD45+ and non-expressing CD3-, CD56-, CD16-, CD19- and CD1c-, as shown in Supplementary Figure 3."

• It would give the work an additional dimension if the authors included more information on the studied molecules in the introduction and discussion sections. For example, describe CD25 as the IL-2 receptor, etc.

We would like to express our gratitude to the reviewer for this valuable suggestion. It is agreed that the provision of additional biological context on the studied molecules would strengthen the manuscript. As a result, we have expanded both the **Introduction** and **Discussion** sections to include concise functional descriptions of the key immune checkpoints analysed in this study (e.g. CD25/IL-2 receptor α, PD-L1, LAG-3, TIM-3), highlighting their immunological roles and their relevance in tumour immune regulation and cancer progression. These additions aim to improve clarity for a broader readership while reinforcing the biological rationale underlying our findings.

Pages 3-4: *In parallel, the immune system plays a decisive role in tumour evolution, and immune checkpoints (ICs) have emerged as key regulators of immune surveillance [14]. Among the sICs analysed in this study, several exert non-redundant roles in the regulation of T-cell activation and immune tolerance. One such element is CD25, which corresponds to the α-chain of the Interleukin (IL)-2 receptor (IL-2rα). This IC is expressed by activated T cells and regulatory T cells, where it governs IL-2-dependent proliferation and immune tolerance [15,16]. In contrast, PD-L1, LAG-3, TIM-3 and CTLA-4 function as co-inhibitory ICs that restrain T-cell activation through complementary mechanisms, including the inhibition of T-cell receptor and co-stimulatory signalling, the promotion of T-cell dysfunction and immune exhaustion, and the maintenance of peripheral tolerance [14,16]. Tumours frequently exploit co-inhibitory pathways*, most notably the PD-L1/PD-1 and CTLA-4 axes, to evade immune detection [17].

Page 10: *Importantly, soluble immune checkpoints are not merely passive biomarkers: they retain biological activity and can actively modulate immune signalling by engaging cognate receptors and ligands [32,44]. Within this context, CTLA-4 represents a central regulator of early T-cell activation, acting upstream of PD-1–mediated inhibition by*

limiting CD28-dependent co-stimulatory signalling. Consistent with its role in the regulation of early T-cell activation, CTLA-4 has been established as a clinically relevant therapeutic target for immune checkpoint blockade, including in lung cancer [45,46].

We would like to express our sincere gratitude to the reviewer for their insightful comments and constructive suggestions, which have greatly contributed to improving the overall quality and clarity of the manuscript.

A Circulating Signature of Tumour Hybrid Cells and Immune Checkpoints Predicts Metastatic Progression in Lung Cancer

Gonzalo Sáenz de Santa María-Diez[1,2,&], Sandra Liana Pardo-Prieto[3,&], Roberto Lozano-Rodríguez[1,2], Urko Aguirre-Larracoechea[4,5,6], María Elena Corpa-Rodríguez[3], Julia del Prado-Montero[1,2], Verónica Terrón-Arcos[1,2], Karla Montalbán-Hernández[1,2], Daniel Arvelo-Rosario[1,2], Jesús Fernández-Felipe[1,2,7], Laura Córdoba[1,9], Gloria C. Bonel-Pérez[1,2], Carlos del Fresno[1,8], Laura Hurtado-Navarro[1,2,7,*] and Eduardo López-Collazo[1,2,7,9,10,*,$]

[1] The Innate Immune Response Group, IdiPAZ, La Paz University Hospital, Madrid, Spain.
[2] Tumour Immunology Laboratory, IdiPAZ, La Paz University Hospital, Madrid, Spain.
[3] Thoracic Surgery Service, La Paz University Hospital.
[4] Research Unit, Osakidetza Basque Health Service, Barrualde-Galdakao Integrated Health Organisation, Galdakao-Usansolo Hospital, Galdakao, Spain.
[5] Kronikgune Institute for Health Services Research, Bilbao, Spain.
[6] Network for Research on Chronicity, Primary Care, and Health Promotion (RICAPPS), Galdakao, Spain.
[7] UNIE University, Madrid. Spain.
[8] Immunomodulation Laboratory, IdiPAZ, La Paz University Hospital, Madrid, Spain
[9] CIBER of Respiratory Diseases (CIBERES), Madrid, Spain.
[10] Biobank Platform, Instituto de Investigación Hospital Universitario La Paz (IdiPAZ), La Paz University Hospital, Madrid, Spain.

[&] These authors contributed equally to this work and are considered co-authors.

*** Correspondence:** EL-C: elopezc@salud.madrid.org, LH-N: laura.hurtado@idipaz.es

[$] **Lead contact**

Keywords: tumour hybrid cells, biomarker, lung cancer, soluble immune checkpoints.

Number of words: 3706

Number of figures: 3

Number of tables: 1

Abstract (198 words):

Lung cancer remains the leading cause of cancer-related mortality worldwide and is frequently diagnosed at advanced stages, when metastatic dissemination is already presented. Tumour hybrid cells (THCs) are rare circulating cells formed through fusion between cancer stem cells with leukocytes, predominately monocytes. These cells combine traits from both lineages, conferring enhanced migratory, invasive and immune-evasive capacities that could promote metastasis. In parallel, soluble immune checkpoints (sICs) have emerged as minimally invasive biomarkers and indicators of systemic immune dysregulation and tumour-driven immune escape. In this study, 31 patients with lung cancer were prospectively enrolled at La Paz University Hospital (Madrid, Spain). Circulating THCs were quantified by spectral flow cytometry, and plasma sICs concentrations were determined using multiplex immunoassays. Patients were stratified by metastatic status and survival. Variables showing the strongest discriminative capacity were integrated into multivariable logistic regression models. Number of THCs, and levels of sCTLA-4, s-41BB, sLAG-3, and sTIM-3 exhibited the strongest discrimination for metastasis, while THCs, sLAG-3, and sTIM-3 distinguished deceased from surviving patients. Integrating predictive models demonstrated high accuracy, and survival analyses supported their prognostic significance. These findings indicate circulating THCs and selected sICs represent promising liquid biomarkers for monitoring lung cancer progression and patient outcomes.

Introduction

Lung cancer (LC) is the second-most commonly diagnosed malignancy worldwide yet remains the leading cause of cancer-related mortality, responsible for nearly two million deaths each year [1]. Tobacco smoking is recognised as a predominant risk factor, contributing to approximately 85% of all lung cancer cases [2]. Despite notable advancements in diagnosis and therapy, most patients are diagnosed at advanced stages, when metastatic dissemination has already occurred [3]. This late detection profoundly limits therapeutic options and contributes to persistently poor overall survival associated with the disease. As with other solid tumours, metastatic spread accounts for more than 90% of LC-related deaths [4].

The biological mechanisms underlying metastasis remain poorly understood. Classical models emphasize the role of circulating tumour cells (CTCs), which detach from the primary lesion, intravasate into the bloodstream, survive immune attack, and colonise distant organs [4,5]. Several theories have been proposed to explain this process. The "seed and soil" hypothesis suggests that metastatic potential depends on the compatibility between tumour cells (the “seed”) and the target organ microenvironment (the “soil”) [6]. The epithelial-to-mesenchymal transition (EMT) model posits that tumour cells acquire migratory and invasive properties through a transient mesenchymal state, later reverting to an epithelial phenotype to colonise new sites [7,8].

An alternative and increasingly supported hypothesis is the cancer–cell fusion theory, first proposed by Otto Aichel in 1911 [5,9]. This model proposes that metastasis may be driven by tumour hybrid cells (THCs), which are heterogeneous cells formed through the fusion of tumour cells with leukocytes, particularly monocytes or macrophages [9,10]. It has been demonstrated that THCs retain essential leukocyte properties such as migration, chemotaxis, and immune evasion, while preserving the proliferative capacity of tumour cells [11]. Experimental evidence supports this hypothesis: THCs has been detected in both primary tumours and peripheral blood [12], and exhibit enhanced migratory and proliferative capacities compared with their parental cells [13]. Transcriptomic studies further suggest that these hybrids overexpress genes involved in motility, immune escape and metabolic adaptation [13]. Collectively, these findings position THCs as both a potential mechanistic driver of metastasis and a promising biomarker for early dissemination and prognosis [5].

In parallel, the immune system plays a decisive role in tumour evolution, and immune checkpoints (ICs) have emerged as key regulators of immune surveillance [14]. Among

the sICs analysed in this study, several exert non-redundant roles in the regulation of T-cell activation and immune tolerance. One such element is CD25, which corresponds to the α-chain of the Interleukin (IL)-2 receptor (IL-2rα). This IC is expressed by activated T cells and regulatory T cells, where it governs IL-2-dependent proliferation and immune tolerance [15,16]. In contrast, PD-L1, LAG-3, TIM-3 and CTLA-4 function as co-inhibitory ICs that restrain T-cell activation through complementary mechanisms, including the inhibition of T-cell receptor and co-stimulatory signalling, the promotion of T-cell dysfunction and immune exhaustion, and the maintenance of peripheral tolerance [14,16]. Tumours frequently exploit co-inhibitory pathways, most notably the PD-L1/PD-1 and CTLA-4 axes, to evade immune detection [17]. Although immune checkpoint blockade (ICB) therapy has revolutionised cancer therapy, eliciting durable responses in certain patients, its efficacy remains limited by resistance mechanisms, heterogeneity in checkpoint expression, immune exhaustion, and the absence of robust predictive biomarkers [18].

Given these challenges, the combined parallel study of tumour hybrid cells and soluble immune checkpoints may offer complementary insights into the intertwined mechanisms of metastasis and immune escape. While THCs may facilitate metastatic spread, sICs can reflect systemic immune dysregulation that promotes tumour persistence [5,14]. The integration of these two biological dimensions could therefore enable the identification of circulating biomarkers with both prognostic and stratification values in lung cancer. Despite the modest sample size and lack of an independent validation cohort, this study underscores the potential of uniting cellular and soluble immune biomarkers to advance our understanding of lung cancer progression and inform precision clinical management.

Results

Description of the lung cancer cohort

To access the prognostic value of soluble immune checkpoints (sICs) and circulating tumour hybrid cells (THCs) in lung cancer (LC), 31 patients were prospectively recruited at the Thoracic Surgery Department of La Paz University Hospital (Madrid, Spain) between 11 March 2020 and 14 April 2023. Circulating THCs and twelve sICs were quantified. Patients were categorised according to key clinical parameters, including tumour histology, anatomical site, TNM classification, disease stage, relapse or progression status, metastatic involvement, prior cancer history, lymphovascular invasion, surgical approach, smoking status, and the presence of comorbid respiratory disease. Most patients were diagnosed at stage I disease, while a minority presented with stage III–IV. The

single-stage IV patient underwent surgical intervention for diagnostic purposes, as prior less-invasive procedures had not yielded a definitive histopathological diagnosis. During the 2.5-year follow-up period, five patients experienced relapse or disease progression. Of these, one case had initially been classified as stage I, one as stage II, two as stage III, and one as stage IV disease. Two of these patients (one stage III and one stage IV) died during the follow-up period. Demographic and clinical characteristics of the cohort are summarised in **Table 1**.

	Total	Alive	*Exitus*	*p* value
**Number of individuals, (%)**	31	28 (84.84)	5 (15.16)	
**Age in years, median (min-max)**	73 (39-85)	72 (39-79)	79 (57-85)	0.3075
**Biological sex, *n* (%)**				
Male	17 (54.83)	13 (76.46)	4 (23.54)	0.2170
Female	14 (45.17)	13 (92.85)	1 (7.15)	
**Tumour type, *n* (%)**				
Squamous-cell carcinoma	8 (25.80)	7 (87.50)	1 (12.50)	0.7459
Adenocarcinoma	23 (74.20)	19 (82.60)	4 (17.40)	
**Primary tumour site, *n* (%)**				
Left upper lobe	13 (41.94)	10 (76.92)	3 (23.07)	
Left lower lobe	4 (12.90)	4 (100)	0 (0)	
Middle lobe	2 (06.45)	2 (100)	0 (0)	0.7489
Right upper lobe	8 (25.81)	7 (23.33)	1 (16.67)	
Right lower lobe	4 (12.90)	3 (75)	1 (25)	
**TNM classification, *n* (%)**				
pT1aN0M0	3 (09.68)	3 (100)	0 (0)	
pT1bN0M0	7 (22.57)	7 (100)	0 (0)	
pT1cN0M0	3 (09.68)	3 (100)	0 (0)	
pT2aN0M0	5 (16.12)	4 (80)	1 (20)	
pT2aN1M0	2 (06.45)	1 (50)	1 (50)	
pT2aN2M0	1 (03.23)	1 (100)	0 (0)	0.3089
pT2bN0M0	2 (06.45)	1 (50)	1 (50)	
pT2aN2M0	3 (09.68)	2 (66.67)	1 (33.32)	
pT3N0M0	2 (06.45)	2 (100)	0 (0)	
pT3N1M1b	1 (03.23)	0 (0)	1 (100)	
pTisN0M0	1 (03.23)	1 (100)	0 (0)	
pTmiN0M0	1 (03.23)	1 (100)	0 (0)	
**Stage, *n* (%)**				
0	1 (03.23)	1 (100)	0 (0)	
I	19 (61.29)	18 (94.74)	1 (5.26)	
II	5 (16.13)	4 (80)	1 (20)	0.0560
III	5 (16.13)	3 (60)	2 (40)	
IV	1 (03.23)	0 (0)	1 (100)	
**Relapse or progression, *n* (%)**				
Yes	5 (16.13)	3 (60)	2 (40)	0.1130
No	26 (83.87)	23 (88.46)	3 (11.56)	

**Metastasis, *n* (%)**				
Yes	5 (16.13)	1 (20)	4 (80)	0.1781
No	26 (83.87)	25 (96.15)	1 (03.85)	
**History of cancer, *n* (%)**				
Yes	11 (33.33)	6 (90.91)	5 (9.09)	0.0010***
No	20 (66.67)	20 (100)	0 (0)	
**Lymphovascular invasion, *n* (%)**				
Yes	2 (6.45)	1 (50)	1 (50)	0.1781
No	29 (93.55)	25 (82.21)	4 (13.79)	
**PD-L1 tissue expression, *n* (%)**				
Not available	4	4 (100)	0 (0)	
< 1 %	11	8 (72.73)	3 (27.27)	0.5861
1-49 %	8	7 (87.5)	1 (22.5)	
≥ 50 %	8	7 (87.5)	1 (22.5)	
**EGFR mutational status, *n* (%)**				
Mutant	7 (22.58)	7 (100)	0 (0)	0.1873
Wild type	24 (77.42)	19 (79.17)	5 (20.83)	
**Systemic treatments, *n* (%)**				
No treatment	13 (41.94)	11 (84.62)	2 (15.38)	
Chemotherapy	11 (35.48)	9 (81.82)	2 (18.18)	0.3522
Radiotherapy	3 (9.68)	3 (100)	0 (0)	
Immunotherapy	4 (12.90)	2 (50)	2 (50)	
**Type of surgery, *n* (%)**				
Segmentectomy	4 (12.90)	4 (100)	0 (0)	0.3473
Lobectomy	27 (87.10)	22 (81.48)	5 (18.52)	
**Smoking status, *n* (%)**				
Non-smoker	5 (16.13)	5 (100)	0 (0)	
Current smoker	5 (16.13)	3 (60)	2 (40)	0.1974
Former smoker	21 (67.74)	19 (90.48)	3 (09.52)	
**Respiratory disease, *n* (%)**				
None	20 (64.52)	18 (90)	2 (10)	
Obstructive sleep apnea	1 (03.22)	1 (100)	0 (0)	0.3379
Chronic obstructive pulmonary disease	10 (32.26)	7 (70)	3 (30)	

Table 1. Clinical and demographic information of the patients included in the study. Statistical analysis was done by Mann-Whitney test and Chi-square test.

Unsupervised data analysis based exclusively on circulating THCs and sICs levels, with no demographic or clinicopathological variables included, identified two distinct clusters of LC patients (**Figure 1A**). To facilitate interpretation of cluster formation, the relative relevance of the individual variables contributing to each cluster, as determined by Graphext, is shown in **Figure 1B**. This analysis revealed differential contributions of soluble immune checkpoint markers between clusters. sPD-1 showed greater relevance in Cluster 2, while sPD-L2 exhibited markedly higher relevance in defining Cluster 1 (**Figure 1B**). To further support this clustering, correlation mapping across all variables further revealed two coherent groups of immunomodulatory factors (**Figure 1C**), suggesting shared regulatory or functional relationships. Notably, sCD25, sCD86, and sTIM-3 showed strong positive correlations, forming a tightly correlated immunomodulatory module, whereas sCTLA-4, sPD-L1, and sLAG-3 grouped within an opposing correlation module (**Figure 1C**).

Immune profiling identifies a four-parameter model associated with metastatic progression in lung cancer

Using the same patient clustering obtained in **Figure 1A**, metastatic status was overlaid to evaluate their distribution across clusters; however, this unsupervised framework did not discriminate metastatic from non-metastatic patients (**Figure 2A**). Survival analyses were restricted to a 2.5-year follow-up period, as all observed deaths occurred within this interval. Among metastatic cases, the single individual located within Cluster 1 exhibited clinical and demographic features comparable to the remaining metastatic patients, except for a greater burden of pre-existing pulmonary disease, including COPD, obstructive sleep apnoea, and prior pulmonary thromboembolism. No additional distinguishing clinical variables were identified.

Direct comparison between metastatic and non-metastatic groups revealed significant higher THC counts in metastatic patients (**Figure 2B**). Among soluble markers, levels of sPD-L1 and sLAG-3 were markedly increased in the metastatic subgroup (**Figure 2C-D**). Conversely, sTIM-3 concentrations were significantly lower in metastatic patients (**Figure 2E**). No significant differences were detected for s4-1BB, sCD25, sCTLA-4, Galectin-9, sCD27, sPD-1, sPD-L2, sSIGLEC5 or sCD86 (**Supplementary Figure 1**).

To further assess the prognostic value of these parameters for metastatic progression, variables with a receiver operating characteristic (ROC) area under the curve (AUC) above 0.7 were included in a binary logistic regression model with backward stepwise selection. This included statistically significant parameters (**Figure 2B-E**), as well as variables with an AUC value above 0.7 that did reach statistical significance (**Supplementary Figure 1A-D**). These variables were considered for their potential to improve the performance of the multivariate model (**Supplementary Figures 1A-C**).

After iterative removal of non-informative predictors, the final model retained five —THC count, CTLA-4, s4-1BB, sTIM-3, and sLAG-3— as the optimal predictors of metastasis. The resulting composite score demonstrated potential discriminatory power, with an optimal cut-off value of -8.399 (Youden index) and an AUC of 0.70 (95% CI: 0.4528–0.9427; p >0.05; **Figure 2F**) with a sensitivity of 80.77% and a specificity of 60% at this threshold. Kaplan-Meier survival analysis confirmed that higher immune scores were significantly associated with reduced metastasis-free survival (**Figure 2G**). Furthermore, a UMAP projection that used only the variables that made up the composite score showed two distinct patients' groups, with metastatic patients appearing more closely distributed than in the initial unsupervised model (**Figure 2H**). Notably, the outlier in this model did not

correspond to that observed in **Figure 2A**, and no consistent clinical or demographic features distinguished the clusters generated.

Predictive modelling of survival based on circulating and soluble immune markers

To explore the prognostic relationship between the circulating THCs, soluble markers, and overall survival, patients were monitored for up to 2.5-years follow-up. The same analytical strategy was applied. As observed for the metastatic subgroups, unsupervised clustering did not differentiate *alive* (*n*=28) from *exitus* (*n*=5), and no clinical or demographic characteristics distinguished non-survivor individuals assigned to the different clusters (**Figure 3A**).

Comparative analysis revealed that sPD-L1 and sLAG-3 levels were significantly elevated in the individuals who deceased (**Figure 3B-C**). THC counts, sTIM-3, and sCD25 approached statistical significance (**Supplementary Figure 2A-C**), with THC counts showing a trend towards higher values in *exitus*, whereas sTIM-3 and sCD25 tended to be higher in alive. No significant differences were observed for sCTLA-4, sSIGLEC5, sCD86, sPD-L2, sCD27, sPD-1, Galectin-9 and s4-1BB (**Supplementary Figure 2D-K**).

Variables exhibiting AUC above 0.7 were included in a binary logistic regression model with backward stepwise selection to identify predictors of survival. The final model incorporated three key parameters —THC count, sLAG-3 and sTIM-3— which collectively defined a survival-associated immune profile (**Figure 3D**). ROC analysis confirmed the robustness of this model, yielding an AUC of 0.8692 (95% CI: 0.7342–1.0000; p<0.01 **; **Figure 3D**). The optimal cut-off value, determined by the Youden index, was −1.421, with a sensitivity of 69.23% and a specificity of 100% at this threshold.

Kaplan-Meier analysis demonstrated that patient with higher composite (*exitus*) score had significantly reduced 2.5 year-survival (**Figure 3E**). UMAP clustering based on this composite score produced three distinct clusters that closely grouped non-survivors in the embedding space (**Figure 3F**).

Discussion

Despite considerable therapeutic advances, lung cancer (LC) remains associated with poor prognosis, largely due to delayed diagnosis, early metastatic dissemination, and therapeutic resistance [19,20]. The introduction of immunotherapy with immune checkpoint inhibitors (ICIs), has improved survival in selected patients; however, a substantial proportion derive limited or no clinical benefit, underscoring the urgent need for robust

prognostic biomarkers capable of capturing tumour aggressiveness and immune escape beyond conventional staging and histopathology [21,22].

The primary objective of this study was to investigate whether circulating immune-related biomarkers, specifically soluble immune checkpoints (sICs) and tumour hybrid cells (THCs), could provide prognostic information in lung cancer. Patients were stratified according to metastatic progression and survival over a 2.5-year follow-up period, enabling the identification of biomarkers associated with both disease dissemination and clinical outcome.

The PD-1/PD-L1 axis represents a central mechanism of tumour-mediated immune evasion [23]. PD-1, also known as CD279, is an inhibitory receptor expressed on activated T cells, B cells, Natural Killer (NK) cells and antigen-presenting cells (APCs), where it limits immune activation [24]. Its ligand, PD-L1 (B7-H1), is induced under inflammatory conditions and is frequently upregulated on tumour cells, allowing them to suppress antitumour T-cell activity through PD-1 engagement [25]. This interaction has been shown to inhibit T-cell proliferation, cytokine release and cytotoxic function, thereby promoting an immunosuppressive microenvironment that favours tumour progression [26]. In lung cancer, PD-L1 expression has been correlated with advanced disease stages, smoking history, and a poor prognosis for patients [27]. In our cohort, soluble PD-L1 (sPD-L1) levels were significantly higher in patients with metastatic disease and in non-survivors. These findings support the notion that circulating PD-L1 reflects a systemic immunosuppressive state associated with aggressive tumour biology. Importantly, the heterogeneous clinical response to PD-1/PD-L1 blockade highlights the limitations of relying on a single immune checkpoint as a prognostic or predictive marker [28,29], reinforcing the value of multiplex immune profiling.

Similarly, soluble LAG-3 (sLAG-3) was markedly increased in metastatic patients and non-survivors. LAG-3 is a key inhibitory receptor expressed on activated T cells and plasmacytoid dendritic cells, where it promotes T-cell exhaustion and immune suppression through engagement with ligands such as FGL1 and Galectin-3 [30–32]. Previous studies have reported LAG-3–positive tumour-infiltrating lymphocytes in both NSCLC and SCLC, often correlating with aggressive tumour features and poor outcome [33,34]. Moreover, lung adenocarcinoma patients with higher numbers of LAG-3^+ positive cells correlated with features of aggressive tumour characteristics [35]. Our findings extend these observations to the systemic level, suggesting that sLAG-3 may serve as a circulating marker of advanced immune dysfunction [31,36].

In contrast to sPD-L1 and sLAG-3, soluble TIM-3 (sTIM-3) was elevated in non-metastatic patients and alive individuals, indicating a distinct immunological role. TIM-3 is expressed across multiple immune compartments and participates in both immune activation and regulation depending on disease stage [37–40]. Notably, recent spatial and single-cell analyses have shown TIM-3 enrichment in early lung adenocarcinoma lesions, suggesting a role in initial immune modulation rather than terminal exhaustion [41]. This contrasts with the case of advanced tumours, where membrane-bound TIM-3 on TILs has been found to correlate with metastasis and poor survival [42,43]. This is consistent with our observation that higher sTIM-3 levels predominated in early-stage, non-metastatic survivors. Together, these findings highlight that the prognostic value of immune checkpoints is context-dependent and stage-specific.

Importantly, soluble immune checkpoints are not merely passive biomarkers: they retain biological activity and can actively modulate immune signalling by engaging cognate receptors and ligands [32,44]. Within this context, CTLA-4 represents a central regulator of early T-cell activation, acting upstream of PD-1–mediated inhibition by limiting CD28-dependent co-stimulatory signalling. Consistent with its role in the regulation of early T-cell activation, CTLA-4 has been established as a clinically relevant therapeutic target for immune checkpoint blockade, including in lung cancer [45,46].

Beyond immune checkpoints, a major strength of this study is the integration of tumour hybrid cells (THCs) into the prognostic framework. We demonstrate that circulating THCs are significantly increased in patients with metastatic disease and in non-survivors, strongly implicating these cells in aggressive clinical behaviour. THCs arise through tumour–leukocyte fusion, a process that confers both malignant traits (genomic instability, proliferative capacity) and leukocyte-derived properties such as enhanced motility, immune evasion, and tissue tropism [5,47,48]. This hybrid phenotype provides a compelling biological explanation for their heightened invasive and metastatic potential.

Consistent with our findings, fusion-derived hybrid cells have been reported in multiple malignancies, including melanoma, colorectal cancer, and breast cancer, where their presence correlates with metastatic spread and poor survival [49–51]. Importantly, THCs have been shown to upregulate immune checkpoints such as PD-L1, SIGLEC5 and CTLA-4, further facilitating immune escape [50,52–54]. The concurrent increase in THCs and immunosuppressive sICs observed in our cohort suggests a relationship between tumour dissemination capacity and systemic immune suppression.

Taken together, our data indicate that THCs and sICs capture two complementary dimensions of tumour progression. THCs reflect the tumour's intrinsic ability to disseminate physically, while sICs could mirror a permissive immunological environment that enables metastatic outgrowth. Their co-detection may therefore define a "pro-metastatic state", characterised by both enhanced migratory competence and impaired immune surveillance.

Although this study is limited by a relatively small cohort size, this limitation is mitigated by the depth of immune profiling and the longitudinal clinical follow-up. The consistency of associations across metastatic status and survival outcomes strengthens the robustness of our conclusions. Importantly, both THCs and sICs can be quantified from peripheral blood, highlighting their potential as minimally invasive biomarkers suitable for repeated monitoring.

From a translational perspective, the integration of THCs and sICs could support personalised medicine by identifying patients at higher risk of early metastasis or poor survival who may benefit from intensified surveillance, combination immunotherapies, or early systemic intervention. Future studies should focus on validating these findings in larger, independent cohorts, integrating THC and sIC profiling with tumour genomics, immune infiltration patterns, and treatment response. Phenotypic and functional characterisation of THCs, alongside longitudinal immune monitoring, will be essential to establish their clinical utility.

Materials and methods

Participant recruitment and eligibility criteria

Peripheral blood samples were obtained from patients with lung cancer (LC, *n* = 31) recruited at the Thoracic Surgery Service of La Paz University Hospital (Madrid, Spain) prior to surgery. All patients had a histologically confirmed diagnosis of LC prior to enrolment, were evaluated in the outpatient clinic, and subsequently reviewed by the hospital's multidisciplinary Lung Tumour Committee.

The inclusion criteria were based on a histopathological diagnosis of primary non-small cell lung cancer (specifically adenocarcinoma and squamous cell carcinoma) in patients requiring diagnostic or therapeutic surgical intervention by the Thoracic Surgery Department at University Hospital La Paz. Furthermore, eligible participants were required to have provided written informed consent for both biobank storage and the collection of peripheral blood samples during routine follow-up prior to surgery.

Patients were excluded from the study if they presented with benign lesions, secondary lung metastases, or pulmonary neuroendocrine tumours. Furthermore, exclusion was applied to patients whose clinical status, such as those in palliative care or elderly patients with mobility issues, prevented sample collection without exceeding the standard of care. Finally, patients receiving postoperative follow-up at external healthcare facilities were excluded to prevent redundant clinical visits and unnecessary blood draws.

Blood samples were collected and processed within 24 hours prior to surgical intervention. Detailed clinical and demographic information for this cohort is provided in **Table 1**.

Clinical definitions

The following clinical definitions were applied to ensure consistent classification of disease status and outcomes throughout the study.

Metastasis was defined as the dissemination of neoplastic cells to organs different from the primary tumour site, as confirmed by clinical and radiological assessment, during follow-up (e.g., lymph nodes, bone, contralateral lung, or other distant organs). Relapse was defined as the reappearance of neoplastic disease after a documented period of clinical, radiological, and/or analytical remission following initial treatment (surgery, chemotherapy, radiotherapy and/or immunotherapy). The progression of the disease was defined as a deterioration in the patient's condition during the follow-up period, as evidenced by the enlargement of existing tumours and/or the emergence of new metastatic lesions, in the absence of a prior interval without disease. The term "exitus" was defined as death from any cause during the follow-up period.

Plasma collection

Plasma was isolated from EDTA-anticoagulated venous blood either by density gradient centrifugation using Ficoll-Plus solution (GE Healthcare BioSciences) or by centrifugation at 3000 rpm for 15 minutes. The plasma fraction was aliquoted and stored at -80ºC until analysis. Prior to use, samples were thawed at 4 °C and clarified by centrifugation at 15,000 *g* for 10 minutes to remove residual cellular debris.

Detection of soluble immune checkpoints

Plasma concentrations of soluble immune checkpoints (sICs) were quantified using the LegendPlex™ (BioLegend, San Diego, CA, USA, catalogue number 740867) following

the manufacturer's instructions. The panel 1 included: sCD25, s4-1BB, sCD27, sCD86, TGF-β1 (free active form), sCTLA-4, sPD-L1, sPD-L2, sPD-1, sTIM-3, sLAG-3, and Galectin-9. Prepared samples were acquired on a FACS Calibur flow cytometer (BD Biosciences), and data were analysed using LegendPlex software (v.8, BioLegend).

ELISA

Soluble SIGLEC5 levels in plasma were measured using a Human SIGLEC5 ELISA kit (Invitrogen, Cat No. EHSIGLEC5) in accordance with the manufacturer's protocol. Absorbance was read at 450 nm using an Epoch™ microplate reader (BioTek, Instruments).

Sample processing and full-spectrum flow cytometry

Whole blood samples were collected in EDTA (ethylenediaminetetraacetic acid anticoagulated) tubes (Vacuette®, Greiner Bio One, Kremsmünster, Austria). Red blood cell lysis was performed using BD PharmLyse™ Lysing Buffer (BD Biosciences), yielding a leukocyte cell suspension, which was washed twice with phosphate-buffered saline (PBS). Cell viability was assessed using LIVE/DEAD™ Fixable Blue dye (Invitrogen). Subsequently, cells were incubated with an antibody panel detailed in **Supplementary Table 1.** The staining panel was designed to measure circulating tumour hybrid cells, gated as the CD14+ population expressing EpCAM+ and CD45+ and non-expressing $CD3^-$, $CD56^-$, $CD16^-$, $CD19^-$ and $CD1c^-$, as shown in **Supplementary Figure 3.**

Statistical analysis and bioinformatic processing

All statistical analyses and graphical representation were performed using GraphPad Prism 10.3.1 (GraphPad Software Inc). Outliers were identified and excluded using the ROUT method ($Q = 1\%$). Data normality was assessed by the Shapiro-Wilk test. Results are presented as violin plots displaying median values and interquartile ranges (IQRs). For two-group comparisons, the Mann-Whitney U test was used in non-parametric data, and the unpaired *t*-test was applied for parametric data.

Binary logistic regression models were constructed using the backward Wald method with IBM SPSS Statistics v.23 software (Armonk, NY). This stepwise approach begins with the full set of candidate independent variables and repeatedly removes them based on their statistical significance. For each variable included in the final model, a corresponding coefficient (B-factor) was calculated to reflect its impact on the differentiation between groups. The final scores were obtained using the following formula: Score =

([Parameter A] x B-factor A) + ([Parameter B] x B-factor B) + ... + ([Parameter X] x B-factor X), as previously described [55,56]. Classifying scores were used to construct receiver operating characteristic (ROC) curves, where the area under the curve (AUC) and optimal cut-off values were obtained as the Youden index. The objective of selecting the most appropriate regression model was to identify a minimal number of variables while ensuring the retention of those exhibiting the greatest statistical significance.

Kaplan-Meier survival analyses were performed using Microsoft Office Excel and Kaplan-Meier Plotter web page (version 2025.06.16). For unsupervised data exploration, including Uniform Manifold Approximation and Projection (UMAP) visualization and hierarchical clustering, Graphext (Madrid, Spain) was employed. Parameters were set as follows: Euclidean metrics, n_neighbours = 30, 127 n_components = 10 and min_dist = 0.01. The number of clusters was automatically determined by the algorithm based on variable strength.

Statistical significance was defined as follows: $p > 0.05$, not significant (*ns*); *, $p < 0.05$; **, $p < 0.01$; ***, $p < 0.001$; and ****, $p < 0.0001$.

Conclusions

This study provides evidence that the combined assessment of circulating tumour hybrid cells and soluble immune checkpoints offers a powerful, minimally invasive approach to prognostic stratification in lung cancer. By capturing both tumour plasticity and immune suppression, this dual biomarker strategy opens new avenues for understanding metastatic progression and tailoring precision oncology strategies.

Clinical Implications

The identification of circulating THCs and sICs as complementary prognostic biomarkers has direct clinical relevance for the management of lung cancer. As both parameters can be quantified from peripheral blood, they offer a minimally invasive approach to risk stratification that may complement conventional staging and tissue-based biomarkers. The co-detection of elevated THCs and immunosuppressive sICs could identify patients in a pro-metastatic state, characterised by enhanced tumour dissemination and impaired immune surveillance, who may benefit from closer clinical monitoring, early systemic intervention, or combination immunotherapeutic strategies. Moreover, integrating THC and sIC profiling into longitudinal patient follow-up may enable dynamic assessment of

disease evolution and treatment response, supporting more personalised and adaptive therapeutic decision-making in lung cancer care.

References

1. Zhou, J.; Xu, Y.; Liu, J.; Feng, L.; Yu, J.; Chen, D. Global Burden of Lung Cancer in 2022 and Projections to 2050: Incidence and Mortality Estimates from GLOBOCAN. *Cancer Epidemiology* **2024**, *93*, 102693, doi:10.1016/j.canep.2024.102693.
2. World Cancer Research - Lung Cancer Available online: https://www.wcrf.org/preventing-cancer/cancer-types/lung-cancer/#what-causes-lung-cancer.
3. González, J.; de Castro, F.; Barrueco, M.; Cordovilla, R.; Fernández, J.; Gómez, F.; Moreno de Vega, B.; Ramos, J.; Serrano, A. Delays in the Diagnosis of Lung Cancer. *Archivos de Bronconeumología* **2003**, *39*, 437–441.
4. Fares, J.; Fares, M.Y.; Khachfe, H.H.; Salhab, H.A.; Fares, Y. Molecular Principles of Metastasis: A Hallmark of Cancer Revisited. *Sig Transduct Target Ther* **2020**, *5*, 28, doi:10.1038/s41392-020-0134-x.
5. López-Collazo, E.; Hurtado-Navarro, L. Cell Fusion as a Driver of Metastasis: Re-Evaluating an Old Hypothesis in the Age of Cancer Heterogeneity. *Front Immunol* **2025**, *16*, 1524781, doi:10.3389/fimmu.2025.1524781.
6. Akhtar, M.; Haider, A.; Rashid, S.; Al-Nabet, A.D.M.H. Paget's "Seed and Soil" Theory of Cancer Metastasis: An Idea Whose Time Has Come. *Adv Anat Pathol* **2019**, *26*, 69–74, doi:10.1097/PAP.0000000000000219.
7. Pastushenko, I.; Brisebarre, A.; Sifrim, A.; Fioramonti, M.; Revenco, T.; Boumahdi, S.; Van Keymeulen, A.; Brown, D.; Moers, V.; Lemaire, S.; et al. Identification of the Tumour Transition States Occurring during EMT. *Nature* **2018**, *556*, 463–468, doi:10.1038/s41586-018-0040-3.
8. Diepenbruck, M.; Christofori, G. Epithelial-Mesenchymal Transition (EMT) and Metastasis: Yes, No, Maybe? *Curr Opin Cell Biol* **2016**, *43*, 7–13, doi:10.1016/j.ceb.2016.06.002.
9. Aichel, O. *Vorträge Und Aufsätze Über Entwickelungsmechanik Der Organismen: Über Zellverschmelzung Mit Qualitativ Abnormer Chromosomenverteilung Als Ursache Der Geschwulstbildung*; 1911;
10. Laberge, G.S.; Duvall, E.; Haedicke, K.; Pawelek, J. Leukocyte–Cancer Cell Fusion—Genesis of a Deadly Journey. *Cells* **2019**, *8*, 170, doi:10.3390/cells8020170.
11. Jiang, C.; Wu, J. Hypothesis: Hematogenous Metastatic Cancer Cells of Solid Tumors May Disguise Themselves as Memory Macrophages for Metastasis. *Front. Oncol.* **2024**, *14*, 1412296, doi:10.3389/fonc.2024.1412296.
12. Whalen, R.M.; Anderson, A.N.; Jones, J.A.; Sims, Z.; Chang, Y.H.; Nederlof, M.A.; Wong, M.H.; Gibbs, S.L. Ultra High Content Analyses of Circulating and Tumor Associated Hybrid Cells Reveal Phenotypic Heterogeneity. *Sci Rep* **2024**, *14*, 7350, doi:10.1038/s41598-024-57381-8.
13. Anderson, A.N.; Conley, P.; Klocke, C.D.; Sengupta, S.K.; Pang, A.; Farley, H.C.; Gillingham, A.R.; Dawson, A.D.; Fan, Y.; Jones, J.A.; et al. Detection of Neoplastic-Immune Hybrid Cells with Metastatic Properties in Uveal Melanoma. *Biomark Res* **2024**, *12*, 67, doi:10.1186/s40364-024-00609-6.
14. He, X.; Xu, C. Immune Checkpoint Signaling and Cancer Immunotherapy. *Cell Res* **2020**, *30*, 660–669, doi:10.1038/s41422-020-0343-4.
15. Shouse, A.N.; LaPorte, K.M.; Malek, T.R. Interleukin-2 Signaling in the Regulation of T Cell Biology in Autoimmunity and Cancer. *Immunity* **2024**, *57*, 414–428, doi:10.1016/j.immuni.2024.02.001.

16. Rezazadeh-Gavgani, E.; Majidazar, R.; Lotfinejad, P.; Kazemi, T.; Shamekh, A. Immune Checkpoint Molecules: A Review on Pathways and Immunotherapy Implications. *Immunity Inflam & Disease* **2025**, *13*, e70196, doi:10.1002/iid3.70196.
17. Wang, P.; Tang, L.; Zhou, B.; Cheng, L.; Zhao, R.C.; Zhang, J. Analytical Methods for the Detection of PD-1/PD-L1 and Other Molecules Related to Immune Checkpoints. *TrAC Trends in Analytical Chemistry* **2022**, *146*, 116505, doi:10.1016/j.trac.2021.116505.
18. Sharma, P.; Goswami, S.; Raychaudhuri, D.; Siddiqui, B.A.; Singh, P.; Nagarajan, A.; Liu, J.; Subudhi, S.K.; Poon, C.; Gant, K.L.; et al. Immune Checkpoint Therapy—Current Perspectives and Future Directions. *Cell* **2023**, *186*, 1652–1669, doi:10.1016/j.cell.2023.03.006.
19. Vicidomini, G. Current Challenges and Future Advances in Lung Cancer: Genetics, Instrumental Diagnosis and Treatment. *Cancers (Basel)* **2023**, *15*, 3710, doi:10.3390/cancers15143710.
20. Lahiri, A.; Maji, A.; Potdar, P.D.; Singh, N.; Parikh, P.; Bisht, B.; Mukherjee, A.; Paul, M.K. Lung Cancer Immunotherapy: Progress, Pitfalls, and Promises. *Mol Cancer* **2023**, *22*, 40, doi:10.1186/s12943-023-01740-y.
21. Wang, J.; Seebacher, N.; Shi, H.; Kan, Q.; Duan, Z. Novel Strategies to Prevent the Development of Multidrug Resistance (MDR) in Cancer. *Oncotarget* **2017**, *8*, 84559–84571, doi:10.18632/oncotarget.19187.
22. Villadolid, J.; Amin, A. Immune Checkpoint Inhibitors in Clinical Practice: Update on Management of Immune-Related Toxicities. *Transl Lung Cancer Res* **2015**, *4*, 560–575, doi:10.3978/j.issn.2218-6751.2015.06.06.
23. Patsoukis, N.; Wang, Q.; Strauss, L.; Boussiotis, V.A. Revisiting the PD-1 Pathway. *Sci. Adv.* **2020**, *6*, eabd2712, doi:10.1126/sciadv.abd2712.
24. Lin, X.; Kang, K.; Chen, P.; Zeng, Z.; Li, G.; Xiong, W.; Yi, M.; Xiang, B. Regulatory Mechanisms of PD-1/PD-L1 in Cancers. *Mol Cancer* **2024**, *23*, 108, doi:10.1186/s12943-024-02023-w.
25. Jeong, H.; Koh, J.; Kim, S.; Yim, J.; Song, S.G.; Kim, H.; Li, Y.; Lee, S.H.; Chung, Y.K.; Kim, H.; et al. Cell-Intrinsic PD-L1 Signaling Drives Immunosuppression by Myeloid-Derived Suppressor Cells through IL-6/Jak/Stat3 in PD-L1-High Lung Cancer. *J Immunother Cancer* **2025**, *13*, e010612, doi:10.1136/jitc-2024-010612.
26. Han, Y.; Liu, D.; Li, L. PD-1/PD-L1 Pathway: Current Researches in Cancer. *Am J Cancer Res* **2020**, *10*, 727–742.
27. Takada, K.; Toyokawa, G.; Okamoto, T.; Shimokawa, M.; Kozuma, Y.; Matsubara, T.; Haratake, N.; Akamine, T.; Takamori, S.; Katsura, M.; et al. A Comprehensive Analysis of Programmed Cell Death Ligand-1 Expression With the Clone SP142 Antibody in Non–Small-Cell Lung Cancer Patients. *Clinical Lung Cancer* **2017**, *18*, 572-582.e1, doi:10.1016/j.cllc.2017.02.004.
28. Guégan, J.-P.; Peyraud, F.; Dadone-Montaudie, B.; Teyssonneau, D.; Palmieri, L.-J.; Clot, E.; Cousin, S.; Roubaud, G.; Cabart, M.; Leroy, L.; et al. Analysis of PD1, LAG3, TIGIT, and TIM3 Expression in Human Lung Adenocarcinoma Reveals a 25-Gene Signature Predicting Immunotherapy Response. *Cell Rep Med* **2024**, *5*, 101831, doi:10.1016/j.xcrm.2024.101831.
29. Davis, A.A.; Patel, V.G. The Role of PD-L1 Expression as a Predictive Biomarker: An Analysis of All US Food and Drug Administration (FDA) Approvals of Immune Checkpoint Inhibitors. *J Immunother Cancer* **2019**, *7*, 278, doi:10.1186/s40425-019-0768-9.

30. Huo, J.-L.; Wang, Y.-T.; Fu, W.-J.; Lu, N.; Liu, Z.-S. The Promising Immune Checkpoint LAG-3 in Cancer Immunotherapy: From Basic Research to Clinical Application. *Front. Immunol.* **2022**, *13*, 956090, doi:10.3389/fimmu.2022.956090.
31. Sauer, N.; Szlasa, W.; Jonderko, L.; Oślizło, M.; Kunachowicz, D.; Kulbacka, J.; Karłowicz-Bodalska, K. LAG-3 as a Potent Target for Novel Anticancer Therapies of a Wide Range of Tumors. *IJMS* **2022**, *23*, 9958, doi:10.3390/ijms23179958.
32. Maruhashi, T.; Sugiura, D.; Okazaki, I.; Okazaki, T. LAG-3: From Molecular Functions to Clinical Applications. *J Immunother Cancer* **2020**, *8*, e001014, doi:10.1136/jitc-2020-001014.
33. Young, H.J.; Pharaon, R.; Bonjoc, K.-J.C.; Ally, F.; Yin, H.; Kang, R.; Gernon, T.; Maghami, E.; Chaudhry, A. Differential Immune Pathways in Classic and Mixed Variants of Anaplastic Thyroid Cancer. *JCO* **2020**, *38*, e18579–e18579, doi:10.1200/JCO.2020.38.15_suppl.e18579.
34. Sun, H.; Dai, J.; Zhao, L.; Zhu, J.; Wang, H.; Chen, P.; Lu, H.; Chen, Q.; Zhang, Z. Lymphocyte Activation Gene-3 Is Associated with Programmed Death-Ligand 1 and Programmed Cell Death Protein 1 in Small Cell Lung Cancer. *Ann Transl Med* **2021**, *9*, 1468–1468, doi:10.21037/atm-21-4481.
35. He, Y.; Yu, H.; Rozeboom, L.; Rivard, C.J.; Ellison, K.; Dziadziuszko, R.; Suda, K.; Ren, S.; Wu, C.; Hou, L.; et al. LAG-3 Protein Expression in Non–Small Cell Lung Cancer and Its Relationship with PD-1/PD-L1 and Tumor-Infiltrating Lymphocytes. *Journal of Thoracic Oncology* **2017**, *12*, 814–823, doi:10.1016/j.jtho.2017.01.019.
36. Workman, C.J.; Wang, Y.; El Kasmi, K.C.; Pardoll, D.M.; Murray, P.J.; Drake, C.G.; Vignali, D.A.A. LAG-3 Regulates Plasmacytoid Dendritic Cell Homeostasis. *J Immunol* **2009**, *182*, 1885–1891, doi:10.4049/jimmunol.0800185.
37. Das, M.; Zhu, C.; Kuchroo, V.K. Tim-3 and Its Role in Regulating Anti-tumor Immunity. *Immunological Reviews* **2017**, *276*, 97–111, doi:10.1111/imr.12520.
38. Sauer, N.; Janicka, N.; Szlasa, W.; Skinderowicz, B.; Kołodzińska, K.; Dwernicka, W.; Oślizło, M.; Kulbacka, J.; Novickij, V.; Karłowicz-Bodalska, K. TIM-3 as a Promising Target for Cancer Immunotherapy in a Wide Range of Tumors. *Cancer Immunol Immunother* **2023**, *72*, 3405–3425, doi:10.1007/s00262-023-03516-1.
39. Zhu, C.; Anderson, A.C.; Schubart, A.; Xiong, H.; Imitola, J.; Khoury, S.J.; Zheng, X.X.; Strom, T.B.; Kuchroo, V.K. The Tim-3 Ligand Galectin-9 Negatively Regulates T Helper Type 1 Immunity. *Nat Immunol* **2005**, *6*, 1245–1252, doi:10.1038/ni1271.
40. Hastings, W.D.; Anderson, D.E.; Kassam, N.; Koguchi, K.; Greenfield, E.A.; Kent, S.C.; Zheng, X.X.; Strom, T.B.; Hafler, D.A.; Kuchroo, V.K. TIM-3 Is Expressed on Activated Human $CD4^+$ T Cells and Regulates Th1 and Th17 Cytokines. *Eur J Immunol* **2009**, *39*, 2492–2501, doi:10.1002/eji.200939274.
41. Zhu, B.; Chen, P.; Aminu, M.; Li, J.-R.; Fujimoto, J.; Tian, Y.; Hong, L.; Chen, H.; Hu, X.; Li, C.; et al. Spatial and Multiomics Analysis of Human and Mouse Lung Adenocarcinoma Precursors Reveals TIM-3 as a Putative Target for Precancer Interception. *Cancer Cell* **2025**, *43*, 1125-1140.e10, doi:10.1016/j.ccell.2025.04.003.
42. Gao, X.; Zhu, Y.; Li, G.; Huang, H.; Zhang, G.; Wang, F.; Sun, J.; Yang, Q.; Zhang, X.; Lu, B. TIM-3 Expression Characterizes Regulatory T Cells in Tumor Tissues and Is Associated with Lung Cancer Progression. *PLoS ONE* **2012**, *7*, e30676, doi:10.1371/journal.pone.0030676.
43. Zang, K.; Hui, L.; Wang, M.; Huang, Y.; Zhu, X.; Yao, B. TIM-3 as a Prognostic Marker and a Potential Immunotherapy Target in Human Malignant Tumors: A

Meta-Analysis and Bioinformatics Validation. *Front. Oncol.* **2021**, *11*, 579351, doi:10.3389/fonc.2021.579351.

44. Zhu, C.; Anderson, A.C.; Schubart, A.; Xiong, H.; Imitola, J.; Khoury, S.J.; Zheng, X.X.; Strom, T.B.; Kuchroo, V.K. The Tim-3 Ligand Galectin-9 Negatively Regulates T Helper Type 1 Immunity. *Nat Immunol* **2005**, *6*, 1245–1252, doi:10.1038/ni1271.
45. Tarhini, A. CTLA-4 Blockade: Therapeutic Potential in Cancer Treatments. *OTT* **2010**, 15, doi:10.2147/OTT.S4833.
46. Oresti, S.; Salomone, F.; Nuccio, A.; Ogliari, F.R.; Riva, S.T.; Mollica, L.; Bulotta, A.; Viganò, M.G.; Venanzi, F.M.; Passaretti, F.; et al. Impact of Platinum-Based Chemotherapy and CTLA-4 Inhibition on Acquired Resistance to First-Line Anti-PD-1/PD-L1 Agents in Non-Small Cell Lung Cancer: A Systematic Review and Reconstructed Individual Patient Data Analysis. *eClinicalMedicine* **2025**, *88*, 103482, doi:10.1016/j.eclinm.2025.103482.
47. Weiler, J.; Dittmar, T. Cell Fusion in Human Cancer: The Dark Matter Hypothesis. *Cells* **2019**, *8*, 132, doi:10.3390/cells8020132.
48. Shultes, P.V.; Weaver, D.T.; Tadele, D.S.; Barker-Clarke, R.J.; Scott, J.G. Cell-Cell Fusion in Cancer: The next Cancer Hallmark? *The International Journal of Biochemistry & Cell Biology* **2024**, *175*, 106649, doi:10.1016/j.biocel.2024.106649.
49. Gast, C.E.; Silk, A.D.; Zarour, L.; Riegler, L.; Burkhart, J.G.; Gustafson, K.T.; Parappilly, M.S.; Roh-Johnson, M.; Goodman, J.R.; Olson, B.; et al. Cell Fusion Potentiates Tumor Heterogeneity and Reveals Circulating Hybrid Cells That Correlate with Stage and Survival. *Sci. Adv.* **2018**, *4*, eaat7828, doi:10.1126/sciadv.aat7828.
50. Aguirre, L.A.; Montalbán-Hernández, K.; Avendaño-Ortiz, J.; Marín, E.; Lozano, R.; Toledano, V.; Sánchez-Maroto, L.; Terrón, V.; Valentín, J.; Pulido, E.; et al. Tumor Stem Cells Fuse with Monocytes to Form Highly Invasive Tumor-Hybrid Cells. *OncoImmunology* **2020**, *9*, 1773204, doi:10.1080/2162402X.2020.1773204.
51. Pawelek, J.M.; Chakraborty, A.K. Fusion of Tumour Cells with Bone Marrow-Derived Cells: A Unifying Explanation for Metastasis. *Nat Rev Cancer* **2008**, *8*, 377–386, doi:10.1038/nrc2371.
52. Montalbán-Hernández, K.; Cantero-Cid, R.; Casalvilla-Dueñas, J.C.; Avendaño-Ortiz, J.; Marín, E.; Lozano-Rodríguez, R.; Terrón-Arcos, V.; Vicario-Bravo, M.; Marcano, C.; Saavedra-Ambrosy, J.; et al. Colorectal Cancer Stem Cells Fuse with Monocytes to Form Tumour Hybrid Cells with the Ability to Migrate and Evade the Immune System. *Cancers* **2022**, *14*, 3445, doi:10.3390/cancers14143445.
53. Tanjak, P.; Chaiboonchoe, A.; Suwatthanarak, T.; Thanormjit, K.; Acharayothin, O.; Chanthercrob, J.; Parakonthun, T.; Methasate, A.; Fischer, J.M.; Wong, M.H.; et al. Tumor-Immune Hybrid Cells Evade the Immune Response and Potentiate Colorectal Cancer Metastasis through CTLA4. *Clin Exp Med* **2024**, *25*, 2, doi:10.1007/s10238-024-01515-9.
54. Tajima, Y.; Shibasaki, F.; Masai, H. Cell Fusion Upregulates PD-L1 Expression for Evasion from Immunosurveillance. *Cancer Gene Ther* **2024**, *31*, 158–173, doi:10.1038/s41417-023-00693-0.
55. Mata-Martínez, P.; Celada, L.; Cueto, F.J.; Sáenz De Santa María, G.; Fernández, J.; Terrón-Arcos, V.; Valdés, N.; Moreira, V.G.; Del Toro, M.I.E.; López-Collazo, E.; et al. A Blood-Based Liquid Biopsy Analyzing Soluble Immune Checkpoints and Cytokines Identifies Distinct Neuroendocrine Tumors. *J Exp Clin Cancer Res* **2025**, *44*, 82, doi:10.1186/s13046-025-03337-3.

56. Del Prado-Montero, J.; Guevara-Martínez, J.; Cantero-Cid, R.; Mata-Martínez, P.; Cueto, F.J.; Lozano-Rodríguez, R.; Terrón-Arcos, V.; Abad-Moret, R.; Díaz-Serrano, E.; De Diego, R.P.; et al. Age-Stratified Circulating Immune Signatures Reveal Non-Invasive Biomarkers for Colorectal Cancer Detection. *Immun Ageing* **2025**, *22*, 40, doi:10.1186/s12979-025-00535-7.

Abbreviations

AUC	Area under the curve
CD	Cluster of differentiation
CTC	Circulating tumour cell
EMT	Epithelial-to-mesenchymal transition
IC	Immune checkpoint
ICI	Immune checkpoint inhibitor
IL	Interleukin
IFN	Interferon
LC	Lung cancer
THC	Tumour hybrid cell
TNF	Tumour necrosis factor

Limitations of the study

It is important to note that the present study is subject to certain limitations, which must be considered when interpreting the results. The cohort size was modest, with 31 patients included, which limits statistical power and may increase the risk of overfitting in the predictive models, particularly in complex models such as those applied in this study. Moreover, the single-centre design restricts the generalisability of the findings, as population characteristics, clinical management, and laboratory conditions may differ across institutions. Another important limitation is the absence of an independent validation cohort, meaning the logistic regression models should be regarded as exploratory and require external confirmation in larger, multicentre datasets. The heterogeneity of tumour histology, clinical stages, comorbidities and treatments within the cohort introduces biological variability that may influence both THC counts and sIC levels, although this reflects real-world clinical practice. Furthermore, all biomarkers were measured at a single pre-surgical time point, thereby preventing the analysis of temporal dynamics or treatment-related changes in THC or sIC profiles. In addition, the cellular sources of the analysed sIC were not characterised, which limits the interpretation of the mechanisms involved, since these circulating factors may originate from multiple tumour and immune cell compartments. Consequently, further mechanistic work is required to elucidate the

biological pathways connecting tumour–immune fusion events with systemic immune dysregulation.

Author contributions

E.L-C and L.H-N designed, conceived and supervised the study. ELC provided fundings. SL.P-P and ME.C-R recruited the study populations. G.SdS, D.A-R, V.T-A, K.M-H, J.dP-M and G. B-P performed the experimental work. G.SdS, L.H-N and E.L-C analysed the data, interpreted results, conceived the experiments, and prepared the figures and paper writing. C.dF, R.L-R, J.F-F, L.C, U.A-L and E.L-C contributed to data interpretation and critical review of the manuscript. All authors have reviewed and edited the output and take full responsibility for the content of this publication.

Funding

This work was funded by the PT20/00004 and PI21/00869 grants to E.L-C (ISCIII, Madrid, Spain) and Fundación Familia Alonso grant to E.L-C. C.dF is funded by Instituto de Salud Carlos III (ISCIII) through the projects CP20/00106, PI21/01178, and PI24/01106 and co-funded by the European Union. L.H-N is funded by Fundación para la Investigación Biomédica Hospital La Paz (FIBHULP–Luis Álvarez 2025) and by Instituto de Salud Carlos III (CD25/00102-Sara Borrell 2025).

Institutional Review Board Statement

The study was conducted in accordance with the Declaration of Helsinki and approved by the Institutional Committee for Human Subjects of La Paz University Hospital (HULP: PI-3521) and the Clinical Research Ethics Committees of the Hospitals of Madrid (17.10.1125-GHM) was provided for all participants.

Informed Consent Statement

Written and informed consent has been obtained from all participants to publish this paper, and all authors have given their approval for the publication of this final version of the manuscript.

Data Availability Statement

No datasets were generated or analysed during the current study. All data relevant to the study are included in the article or uploaded as supplementary information and are available from the corresponding author on reasonable request.

Acknowledgements

We would like to thank the entire research team of the Immune Response group and the Tumour Immunology laboratory at IdiPAZ for comments and suggestions thought the development of this project.

Conflicts of Interest

L.H.-N. is co-founder and have shares in Viva *in vitro* diagnostics SL but declare that the research was conducted in the absence of any commercial or financial relationships that could be construed as a potential conflict of interest. The remaining authors declare no competing interests.

Figure legends

Figure 1. Correlation network and clustering of THCs and soluble ICs in patients with lung cancer. (**A**) Unsupervised clustering of lung cancer cohort (*n*= 31) based on all experimental variables analysed (sICs and circulating THCs). (**B**) Relative relevance of circulating THCs and sICs variables contributing to clusters definition. (**C**) Correlation matrix illustrating Pearson correlation coefficients between the levels of individual and the presence of circulating THCs. The colour scale represents the strength and direction of correlation (red: negative; blue: positive correlation), and clusters are delineated in red.

Figure 2. Multivariate predictive modelling of metastasis in patients with lung cancer.

(**A**) Unsupervised UMAP representation of the of lung cancer cohort stratified by metastatic status (metastatic: dark orange, non-metastatic: light orange) using all experimental variables (sICs and THCs). Dashed lines correspond to clusters identified in **Figure 1A**. (**B-E**) Circulating THC counts (**B**) and plasma levels of sPD-L1 (**C**), sLAG-3 (**D**), sTIM-3 (**E**) in lung cancer patients categorised as non-metastatic and metastatic. The right panels of (**B–E**) show receiver operating characteristic (ROC) curves for each individual parameter with the corresponding area under the curves (AUC) and their 95% confidence intervals (CI). Parameters meeting both suggestive statistical significance ($p \leq 0.20$) and acceptable discriminatory performance ($AUC \geq 0.70$) were retained as potential predictors. (**F**) These variables were included in a binary logistic regression model (Backward Wald method) to generate a composite metastasis score. The optimal classification threshold, defined by the Youden index, is indicated by the dashed line. The

right panel of (**F**) displays the ROC curve assessing the predictive accuracy of the model. (**G**) Kaplan–Meier survival analysis according to the metastasis score. (**H**) Clustering of lung cancer patients based to their metastasis score, coloured by metastasis status (metastatic: dark orange, non-metastatic: light orange).

In the left panels of (**B-F**), each dot represents an individual patient. Data are presented as violin plots; statistical comparison between groups were performed using the Mann-Whitney *t*-test was used to compare the difference between groups. Abbreviations: AUC; area under the curve, CI; confidence interval. Statistical significance: ** $p < 0.01$ and * $p < 0.05$.

Figure 3. Predictive modelling of survival outcomes in patients with lung cancer.

(**A**) Unsupervised UMAP representation of the lung cancer cohort categorised by mortality (*exitus*: dark grey, alive: light blue) using all experimental variables (sICs and THCs). The dashed lines correspond to identified in **Figure 1A**. (**B-C**) Plasma levels of s-L1 (**B**) and sLAG-3 (**C**) in lung cancer patients. The right panels of (**B–C**) display ROC curves for each parameter with their respective AUC values and their 95% CI. Parameters showing both suggestive statistical significance (p = ≤ 0.05 to 0.2) and strong discriminatory ability (AUC ≥ 0.7) were selected as candidate predictors. (**D**) These variables were incorporated into a binary logistic regression model (Backward Wald method) to derive a composite mortality score. The optimal classification threshold determined by the Youden index is indicated by the dashed line. The right panel of (**D**) shows the ROC curve assessing model performance. (**E**) Kaplan–Meier survival analysis according to the mortality score. (**F**) Clustering of lung cancer patients based on the mortality score, coloured by survival status (*exitus*: dark grey; alive: light blue). In the left panels of (**B-D**), each dot represents an individual patient. Data are displayed as violin plots; statistical comparison between groups were performed using the Mann-Whitney *t*-test. Abbreviations: AUC; area under the curve, CI; confidence interval. Statistical significance: ** $p < 0.01$ and * $p < 0.05$.

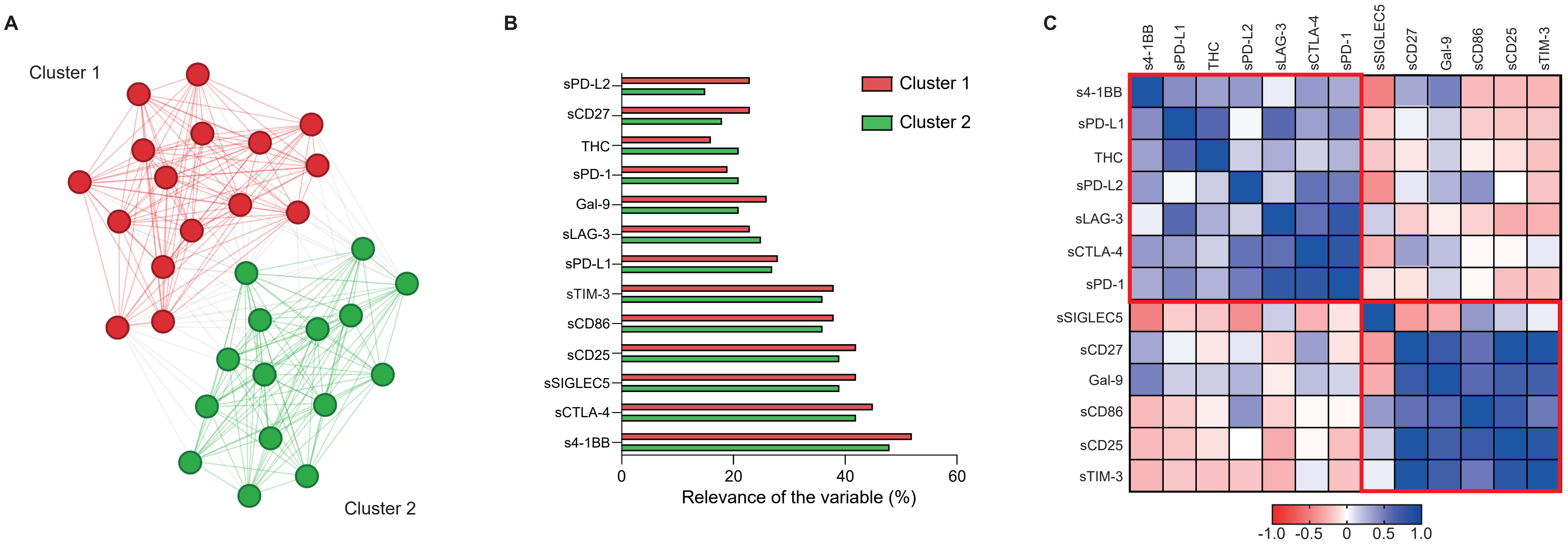
A
Cluster 1
Cluster 2
B
sPD-L2
sCD27
THC
sPD-1
Gal-9
sLAG-3
sPD-L1
sTIM-3
sCD86
sCD25
sSIGLEC5
sCTLA-4
s4-1BB
Cluster 1
Cluster 2
0
20
40
60
Relevance of the variable (%)
C
s4-1BB
sPD-L1
THC
sPD-L2
sLAG-3
sCTLA-4
sPD-1
sSIGLEC5
sCD27
Gal-9
sCD86
sCD25
sTIM-3
-1.0 -0.5 0 0.5 1.0

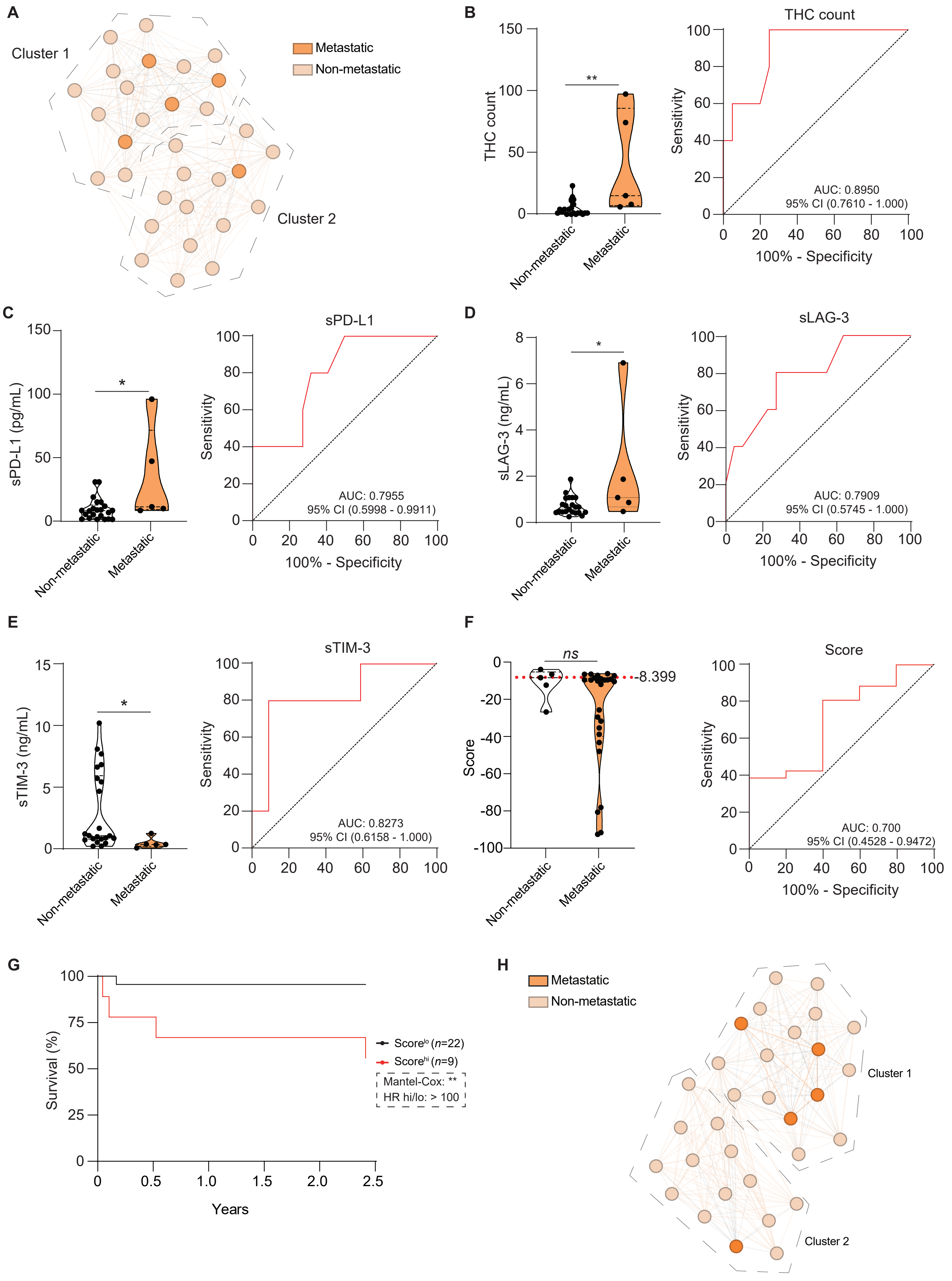
A
Cluster 1
Metastatic
Non-metastatic
Cluster 2
B
THC count
**
Non-metastatic
Metastatic
Sensitivity
AUC: 0.8950
95% CI (0.7610 - 1.000)
100% - Specificity
C
sPD-L1 (pg/mL)
*
sPD-L1
AUC: 0.7955
95% CI (0.5998 - 0.9911)
D
sLAG-3 (ng/mL)
sLAG-3
AUC: 0.7909
95% CI (0.5745 - 1.000)
E
sTIM-3 (ng/mL)
sTIM-3
AUC: 0.8273
95% CI (0.6158 - 1.000)
F
ns
-8.399
Score
AUC: 0.700
95% CI (0.4528 - 0.9472)
G
Survival (%)
Years
Score^lo (n=22)
Score^hi (n=9)
Mantel-Cox: **
HR hi/lo: > 100
H

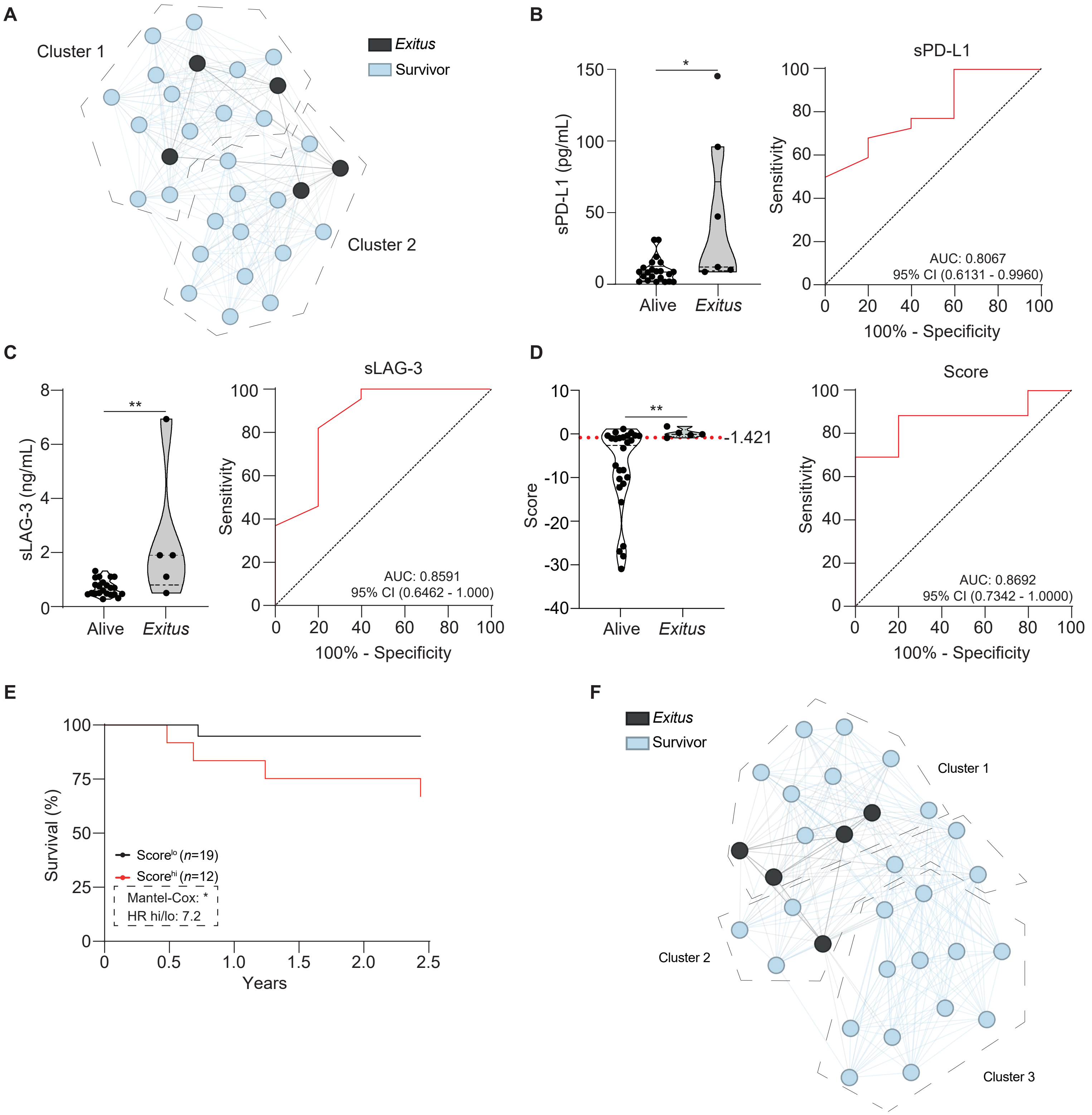
A
Cluster 1
Cluster 2
Exitus
Survivor
B
sPD-L1 (pg/mL)
150
100
50
0
*
Alive
Exitus
sPD-L1
Sensitivity
100% - Specificity
AUC: 0.8067
95% CI (0.6131 - 0.9960)
C
sLAG-3 (ng/mL)
**
Alive
Exitus
sLAG-3
Sensitivity
100% - Specificity
AUC: 0.8591
95% CI (0.6462 - 1.000)
D
Score
**
-1.421
Alive
Exitus
Score
Sensitivity
100% - Specificity
AUC: 0.8692
95% CI (0.7342 - 1.0000)
E
Survival (%)
Years
Score^lo (n=19)
Score^hi (n=12)
Mantel-Cox: *
HR hi/lo: 7.2
F
Exitus
Survivor
Cluster 1
Cluster 2
Cluster 3

Supplementary Figures

(Sáenz de Santa María-Diez *et al*.)

Supplementary Figure 1. Evaluation of circulating soluble immune checkpoints (ICs) according to metastatic status in healthy volunteers (HVs) and Lung Cancer (LC) patients. Plasma levels of (**A**) s4-1BB, (**B**) sCD25, (**C**) Galectin-9, (**D**) sCTLA-4, (**E**) sCD27, (**F**) sPD-1, (**G**) sPD-L2, (**H**) sSIGLEC5 and (**I**) sCD86 of LC patients and HVs according to their metastatic status (metastatic *versus* non-metastatic). The right panels of (**A-D**) show receiver operating characteristic (ROC) curves for each individual parameter with the corresponding area under de curves (AUC) and their 95% confidence intervals (CI). Parameters meeting both suggestive statistical significance ($p \leq 0.20$) and acceptable discriminatory performance (AUC ≥ 0.70) were retained as potential predictors. In the left panels of (**A-D**) and in panels (**E-I**), each dot represents an individual patient. Data are presented as violin plots; statistical comparison between groups were performed using the Mann-Whitney *t*-test was used to compare the difference between groups. Abbreviations: AUC; area under the curve, CI; confidence interval.

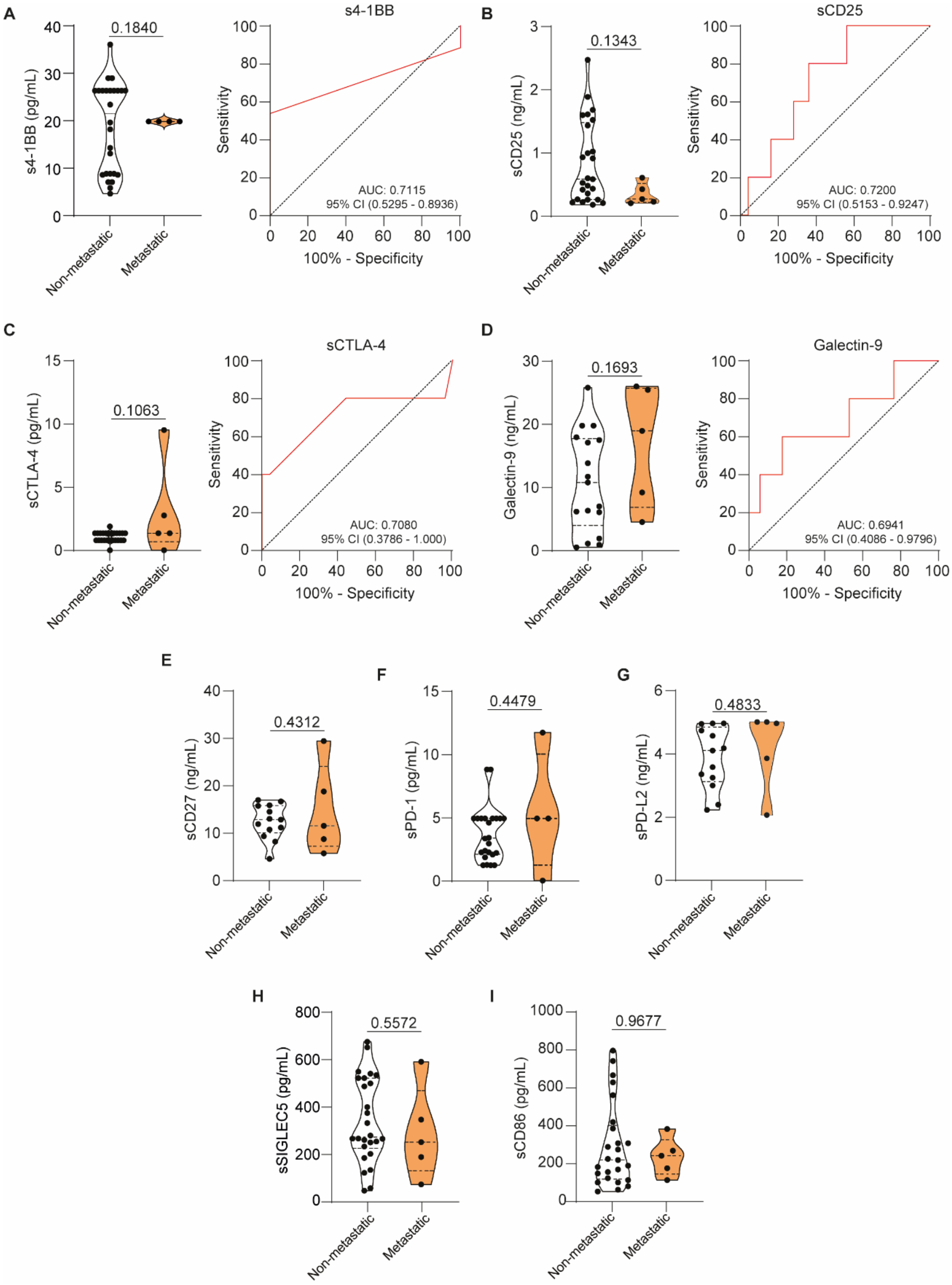
A
s4-1BB (pg/mL)
0.1840
Non-metastatic
Metastatic
s4-1BB
Sensitivity
100% - Specificity
AUC: 0.7115
95% CI (0.5295 - 0.8936)
B
sCD25 (ng/mL)
0.1343
Non-metastatic
Metastatic
sCD25
Sensitivity
100% - Specificity
AUC: 0.7200
95% CI (0.5153 - 0.9247)
C
sCTLA-4 (pg/mL)
0.1063
Non-metastatic
Metastatic
sCTLA-4
Sensitivity
100% - Specificity
AUC: 0.7080
95% CI (0.3786 - 1.000)
D
Galectin-9 (ng/mL)
0.1693
Non-metastatic
Metastatic
Galectin-9
Sensitivity
100% - Specificity
AUC: 0.6941
95% CI (0.4086 - 0.9796)
E
sCD27 (ng/mL)
0.4312
Non-metastatic
Metastatic
F
sPD-1 (pg/mL)
0.4479
Non-metastatic
Metastatic
G
sPD-L2 (ng/mL)
0.4833
Non-metastatic
Metastatic
H
sSIGLEC5 (pg/mL)
0.5572
Non-metastatic
Metastatic
I
sCD86 (pg/mL)
0.9677
Non-metastatic
Metastatic

Supplementary Figure 2. Evaluation of circulating soluble immune checkpoints (ICs) according to survival status in healthy volunteers (HVs) and Lung Cancer (LC) patients. Circulating THCs (**A**) and plasma levels of (**B**) sTIM-3, (**C**) sCD25, (**D**) sCTLA-4, (**E**) sSIGLEC5, (**F**) sCD86, (**G**) sPD-L2, (**H**) sCD27, (**I**) sPD-1, (**J**) Galectin-9 and (**K**) s4-1BB of LC patients and HVs according to their survival status (alive *versus exitus*). The right panels of (**A-C**) show receiver operating characteristic (ROC) curves for each individual parameter with the corresponding area under de curves (AUC) and their 95% confidence intervals (CI). Parameters meeting both suggestive statistical significance ($p \leq 0.20$) and acceptable discriminatory performance (AUC ≥ 0.70) were retained as potential predictors. In the left panels of (**A-C**) and in panels (**D-I**), each dot represents an individual patient. Data are presented as violin plots; statistical comparison between groups were performed using the Mann-Whitney *t*-test was used to compare the difference between groups. Abbreviations: AUC; area under the curve, CI; confidence interval.

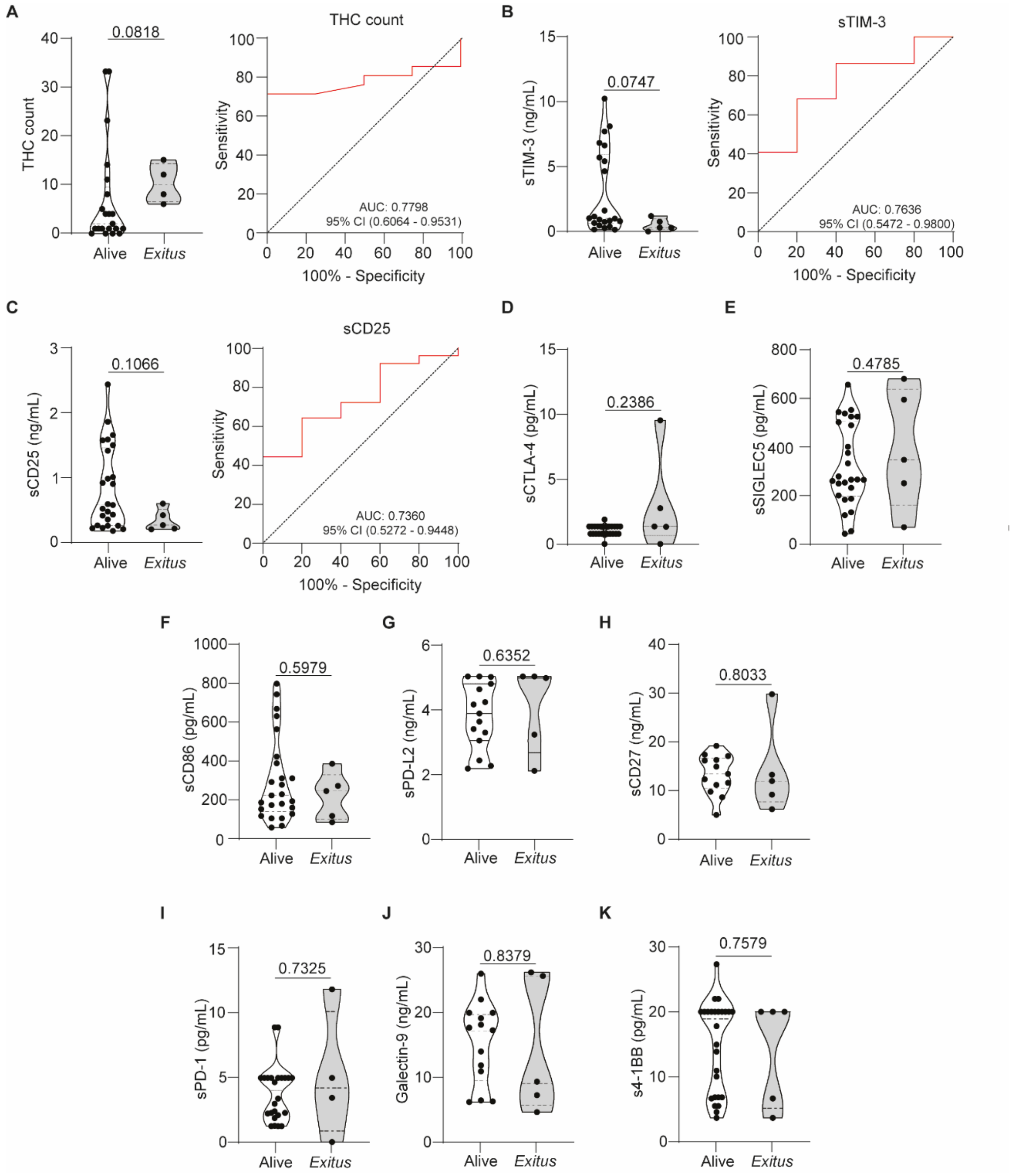
A
B
C
D
E
F
G
H
I
J
K
THC count
0.0818
Alive
Exitus
Sensitivity
100% - Specificity
AUC: 0.7798
95% CI (0.6064 - 0.9531)
sTIM-3 (ng/mL)
0.0747
sTIM-3
AUC: 0.7636
95% CI (0.5472 - 0.9800)
sCD25 (ng/mL)
0.1066
sCD25
AUC: 0.7360
95% CI (0.5272 - 0.9448)
sCTLA-4 (pg/mL)
0.2386
sSIGLEC5 (pg/mL)
0.4785
sCD86 (pg/mL)
0.5979
sPD-L2 (ng/mL)
0.6352
sCD27 (ng/mL)
0.8033
sPD-1 (pg/mL)
0.7325
Galectin-9 (ng/mL)
0.8379
s4-1BB (pg/mL)
0.7579

Supplementary Figure 3. Tumour Hybrid Cells (THCs) gating strategy. Initial events were gated based on size (FSC-H) and granularity (SSC-H). The exclusion of doublets was achieved through the utilization of consecutive pulse-processing channels, whereby the height was plotted against the area for both side scatter (SSC-A/SSC-H) and forward scatter (FSC-A/FSC-H). The viability of cells was determined by the exclusion of a Fixable Viability Dye (L/D), and the subsequent identification of total leukocytes was achieved by CD45 expression. In the CD45+ population, a lineage exclusion strategy was employed to eradicate $CD3^+$, $CD19^+$, $CD56^+$, $CD1c^+$ and $CD16^+$ (T cells, B cells, NK cells and dendritic cells). Within the remaining negative population, monocyte subsets were defined by $CD14^+$ expression. The final plot demonstrates the targeted analysis of the epithelial marker $EpCAM^+$ within the CD14+ monocyte fraction (THCs cells). Arrows and encircled letters indicate the origin of each plot.

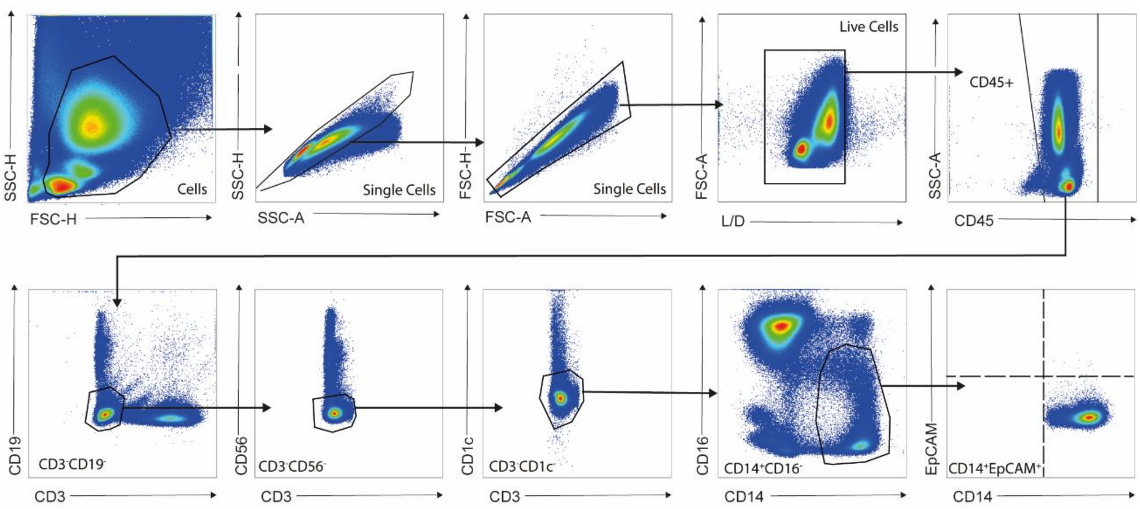

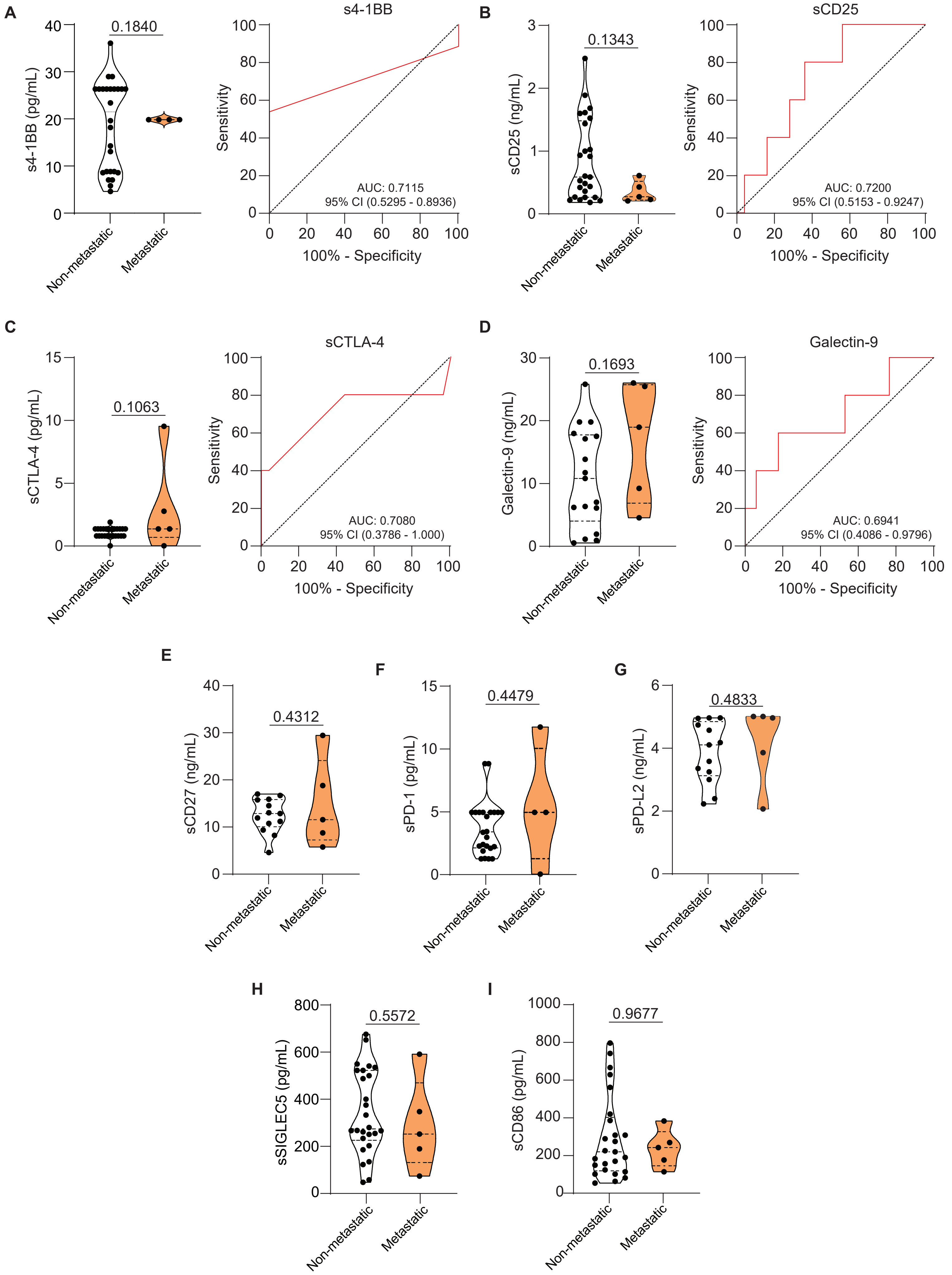

A
s4-1BB (pg/mL)
0.1840
Non-metastatic
Metastatic
s4-1BB
Sensitivity
100% - Specificity
AUC: 0.7115
95% CI (0.5295 - 0.8936)
B
sCD25 (ng/mL)
0.1343
Non-metastatic
Metastatic
sCD25
Sensitivity
100% - Specificity
AUC: 0.7200
95% CI (0.5153 - 0.9247)
C
sCTLA-4 (pg/mL)
0.1063
Non-metastatic
Metastatic
sCTLA-4
Sensitivity
100% - Specificity
AUC: 0.7080
95% CI (0.3786 - 1.000)
D
Galectin-9 (ng/mL)
0.1693
Non-metastatic
Metastatic
Galectin-9
Sensitivity
100% - Specificity
AUC: 0.6941
95% CI (0.4086 - 0.9796)
E
sCD27 (ng/mL)
0.4312
Non-metastatic
Metastatic
F
sPD-1 (pg/mL)
0.4479
Non-metastatic
Metastatic
G
sPD-L2 (ng/mL)
0.4833
Non-metastatic
Metastatic
H
sSIGLEC5 (pg/mL)
0.5572
Non-metastatic
Metastatic
I
sCD86 (pg/mL)
0.9677
Non-metastatic
Metastatic

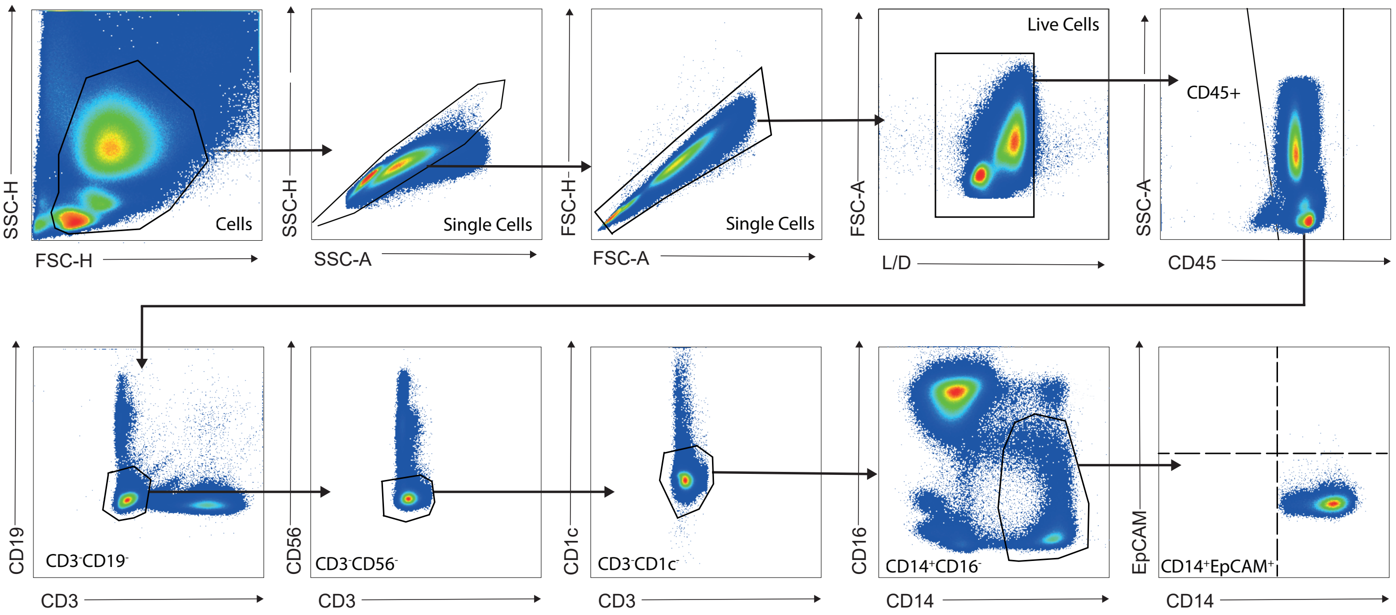

SSC-H
FSC-H
Cells
SSC-H
SSC-A
Single Cells
FSC-H
FSC-A
Single Cells
FSC-A
L/D
Live Cells
SSC-A
CD45
CD45+
CD19
CD3
CD3-CD19-
CD56
CD3
CD3-CD56-
CD1c
CD3
CD3-CD1c-
CD16
CD14
CD14+CD16-
EpCAM
CD14
CD14+EpCAM+

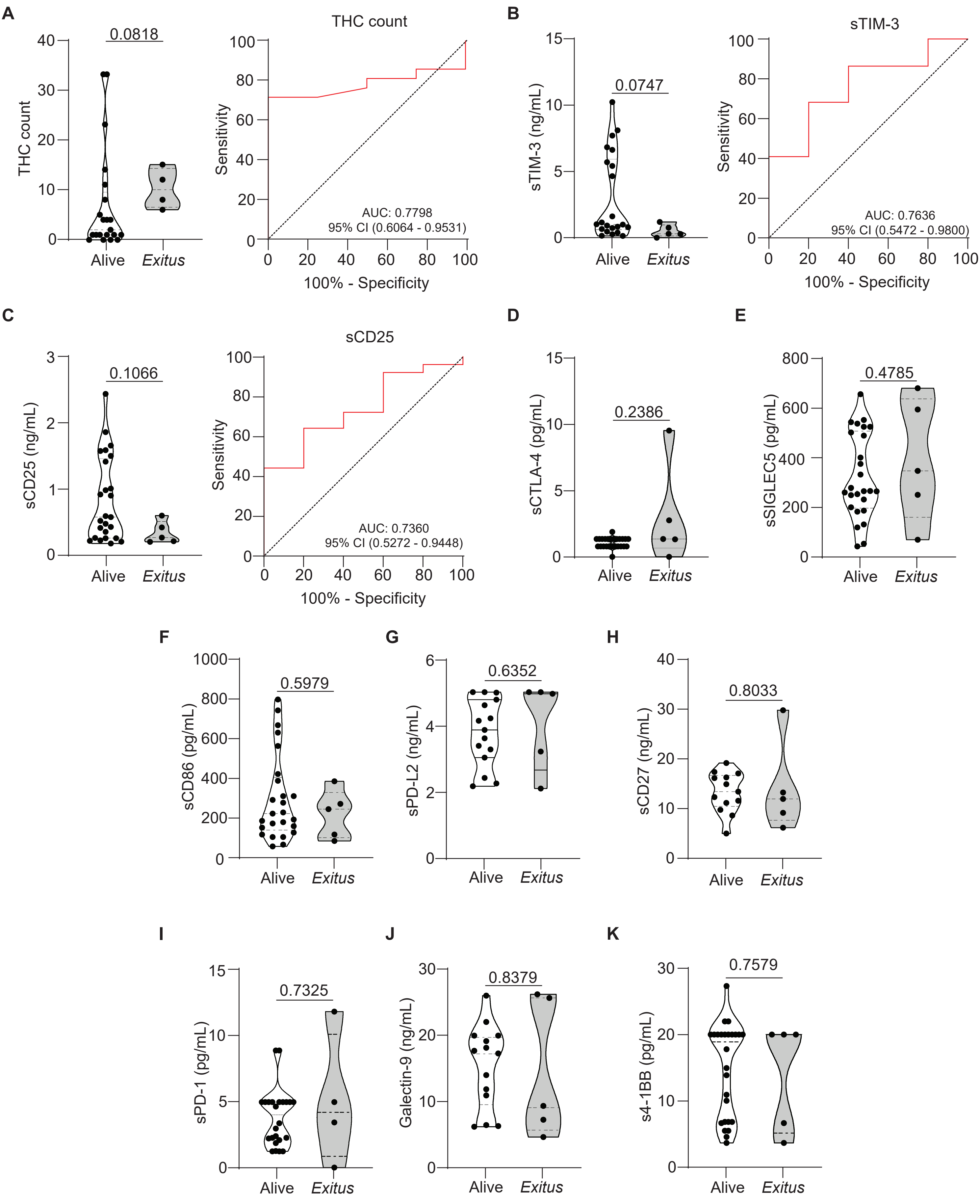

A
THC count
0.0818
Alive
Exitus
THC count
Sensitivity
100% - Specificity
AUC: 0.7798
95% CI (0.6064 - 0.9531)
B
sTIM-3 (ng/mL)
0.0747
Alive
Exitus
sTIM-3
Sensitivity
100% - Specificity
AUC: 0.7636
95% CI (0.5472 - 0.9800)
C
sCD25 (ng/mL)
0.1066
Alive
Exitus
sCD25
Sensitivity
100% - Specificity
AUC: 0.7360
95% CI (0.5272 - 0.9448)
D
sCTLA-4 (pg/mL)
0.2386
Alive
Exitus
E
sSIGLEC5 (pg/mL)
0.4785
Alive
Exitus
F
sCD86 (pg/mL)
0.5979
Alive
Exitus
G
sPD-L2 (ng/mL)
0.6352
Alive
Exitus
H
sCD27 (ng/mL)
0.8033
Alive
Exitus
I
sPD-1 (pg/mL)
0.7325
Alive
Exitus
J
Galectin-9 (ng/mL)
0.8379
Alive
Exitus
K
s4-1BB (pg/mL)
0.7579
Alive
Exitus

Supplementary Table

(Sáenz de Santa María-Diez *et al*.)

Supplementary Table 1. Panel of fluorochrome-conjugated monoclonal antibodies used in flow cytometry for immune profiling.

Marker	Fluorochrome	Source	Clone	Catalog number
CD45	PO	Invitrogen	HI30	MHCD4530
CD16	BV480	BD	3G8	566108
CD14	BV315	BD	MΦP9	563561
CD3	PerCP	Biolegend	SK7	344814
CD19	BV786	BD	SJ25C1	563325
CD56	BUV737	BD	NCAM16.2	612766
CD1c	SB436	BioSciences	L161	62-0015-42
EpCAM	BV605	BD	EBA-1	563182
Siglec5	APC	Biolegend	1ª5	352006

PO, Pacific-Orange; BV, Brilliant Violet; BUV, Brilliant Ultra Violet; PerCP, Phycoerythrin; APC, Allophycocyanin; SB, Super Bright.
